# Multikinase Inhibition with Tinengotinib in Advanced Solid Tumors: Results from a Multicenter Phase Ib/II Trial

**DOI:** 10.1158/1078-0432.CCR-26-1076

**Published:** 2026-07-23

**Authors:** Sarina A. Piha-Paul, Chih-Yi Liao, Farshid Dayyani, Nashat Y. Gabrail, Syed Mohammad Ali Kazmi, Sayeh Moazami Lavasani, Katie Hennessy, Shumao Ni, Caixia Sun, Hui Wang, Peng Peng, Frank Wu, Milind M. Javle, Jean Fan, Sanjay Goel

**Affiliations:** 1 Division of Cancer Medicine, Department of Investigational Cancer Therapeutics, The University of Texas MD Anderson Cancer Center, Houston, Texas.; 2 Hematology and Oncology, Department of Medicine, University of Chicago, Chicago, Illinois.; 3 Division of Hematology/Oncology, Department of Medicine, University of California Irvine, Orange, California.; 4 Department of Oncology, Gabrail Cancer Center, Canton, Ohio.; 5 Division of Hematology and Oncology, University of Texas Southwestern Medical Center, Dallas, Texas.; 6 Department of Medical Oncology and Therapeutics Research, City of Hope Comprehensive Cancer Center, Duarte, California.; 7 TransThera Sciences (US), Inc., Gaithersburg, Maryland.; 8 TransThera Sciences (Nanjing), Inc., Nanjing, China.; 9 Division of Cancer Medicine, Department of Gastrointestinal Medical Oncology, The University of Texas MD Anderson Cancer Center, Houston, Texas.; 10 Department of Medical Oncology, Rutgers Cancer Center Institute, New Brunswick, New Jersey.

## Abstract

**Purpose:**

Tinengotinib, a multikinase inhibitor targeting Aurora kinases A/B, fibroblast growth factor receptors (FGFRs) 1 to 3, vascular endothelial growth factor receptor 2, and Janus kinases 1 to 2, was evaluated in this phase Ib/II study to assess safety, pharmacokinetics (PK), efficacy, and genomic correlates of response and resistance in patients with advanced solid tumors.

**Patients and Methods:**

Patients with advanced/metastatic solid tumors lacking standard treatment options were enrolled at 7 sites in the United States (NCT04742959). Tinengotinib was administered at 12 mg once daily in arm A and at multiple dose levels/dosing schedules in arm C.

**Results:**

Among 197 patients receiving tinengotinib, treatment-related treatment-emergent adverse events occurred in 160 (81.2%) patients, with hypertension being the most common. Hyperphosphatemia and ocular toxicities were uncommon. Clinical benefit was achieved by 43 of 159 (27.0%) evaluable patients, including 1 complete response, 20 partial responses, and 22 stable diseases ≥6 months. Antitumor activity was most pronounced with 10-mg and 12-mg dose levels. The objective response rates were 33.3%, 30%, and 13% in prostate cancer (*n* = 15), breast cancer (*n* = 20), and cholangiocarcinoma (*n* = 23), respectively. PK analyses supported the 10-mg once-daily regimen. Exploratory genomic analyses identified potential molecular correlations of response, including *FGFR2* fusions in cholangiocarcinoma responders. Additional observations in breast cancer, including *ROS1* alterations, were exploratory and should be considered hypothesis generating. Exploratory analyses also suggested associations between *EGFR* or *MLL* mutations in cholangiocarcinoma and *MYC* alterations in prostate cancer with shorter progression-free survival, consistent with emerging evidence linking *MYC*-driven lineage plasticity to androgen receptor–independent disease biology. Reductions in circulating tumor DNA variant allele frequency further supported pharmacodynamic target engagement.

**Conclusions:**

Tinengotinib demonstrated manageable toxicity with antitumor activity across advanced solid tumors, with infrequent hyperphosphatemia and ocular toxicities. Integrated PK–biomarker analyses support the continued clinical development of tinengotinib and the exploration of biomarker-informed precision oncology strategies.

**Translational Relevance**

Resistance and class-associated toxicities limit the clinical benefit of selective fibroblast growth factor receptor (FGFR) inhibitors in biomarker-defined cancers. Tinengotinib, a multikinase inhibitor targeting FGFRs 1 to 3, Aurora kinases, vascular endothelial growth factor receptor 2, and Janus kinase signaling pathways, has the potential to overcome these limitations. In this multicenter phase Ib/II study of patients with advanced solid tumors, tinengotinib demonstrated manageable toxicity and antitumor activity across multiple tumor types, with a relatively low incidence of class-associated FGFR toxicities compared with selective FGFR inhibitors. Exploratory biomarker analyses identified genomic correlates of response, including *FGFR2* fusions in cholangiocarcinoma and *ROS1* mutations in breast cancer, whereas *MYC* alterations in prostate cancer were associated with shorter progression-free survival, consistent with emerging evidence that *MYC*-driven transcriptional reprogramming may facilitate lineage plasticity and transition toward androgen receptor–independent/neuroendocrine disease states. Pharmacodynamic target engagement was further supported by reductions in circulating tumor DNA variant allele frequency during treatment. These findings support the continued clinical development of tinengotinib and highlight the potential for biomarker-informed precision oncology strategies.

## Introduction

Dysregulated kinase signaling networks drive tumor proliferation, angiogenesis, and therapeutic resistance across many malignancies, making kinase inhibition a central strategy in modern oncology (1, 2). However, responses to highly selective kinase inhibitors are often limited by adaptive resistance mechanisms, including acquired mutations, activation of compensatory signaling pathways, and lineage plasticity programs (3, 4).

Tinengotinib is a multikinase inhibitor designed to target pathways involved in tumor proliferation, angiogenesis, and immune regulation. Tinengotinib inhibits Aurora kinases A/B, fibroblast growth factor receptors (FGFRs) 1 to 3, vascular endothelial growth factor receptor 2 (VEGFR2), and Janus kinases (JAK) 1 and 2 (5, 6). Through simultaneous inhibition of these signaling pathways, tinengotinib has the potential to suppress oncogenic signaling networks and adaptive resistance mechanisms that limit the activity of highly selective targeted therapies. In preclinical studies, tinengotinib demonstrated antitumor activities across multiple cancer models, including triple-negative breast cancer (6), and showed favorable pharmacokinetic (PK) properties and oral bioavailability (7).

Results from a phase I study of tinengotinib (*N* = 48) demonstrated preliminary clinical activity in several tumor types, including prostate cancer, hormone receptor (HR)–positive/human epidermal growth factor receptor 2 (HER2)–negative breast cancer, triple-negative breast cancer, and cholangiocarcinoma (5). The most common treatment-related treatment-emergent adverse event (TEAE) was hypertension (8). In cholangiocarcinoma, patients harboring *FGFR* fusions have demonstrated clinical benefit with selective FGFR inhibitors; however, disease progression frequently occurs within months of treatment initiation (9). Secondary mutations in the FGFR2 kinase domain have been identified as an important mechanism of acquired resistance to FGFR inhibitors (9). Tinengotinib was designed to address these resistance mechanisms and has demonstrated activity against FGFR2 kinase domain mutations in preclinical models (9, 10).

The objective of this phase Ib/II trial was to assess the safety, PK profile, preliminary antitumor activity, and exploratory biomarker profiles of tinengotinib monotherapy in patients with locally advanced or metastatic solid tumors lacking standard treatment options.

## Patients and Methods

### Study design and procedures

This phase Ib/II, multicenter, open-label study evaluated the safety and preliminary efficacy of oral tinengotinib as monotherapy or in combination with nab-paclitaxel in patients with advanced or metastatic solid tumors (ClinicalTrials.gov identifier: NCT04742959). Patients were enrolled at 7 locations in the United States and followed for an average of 9 months. Eligible patients were assigned to receive tinengotinib 12 mg once daily for solid tumors (arm A); tinengotinib 8, 10, or 12 mg once daily in combination with 100 mg/m^2^ nab-paclitaxel for metastatic HER2-negative breast cancer (arm B); or tinengotinib 5, 8, 10, or 12 mg once daily or 4 or 6 mg twice daily for solid tumors (arm C). The results for the monotherapy dosage groups of arms A and C are reported in this study; results for arm B will be published separately.

Tinengotinib was supplied as 4-, 5-, and 6-mg tablets and dosed on a flat scale of milligrams per day. For arm A, 12 mg once daily was orally administered on days 1 to 21 in the first 28-day treatment cycle and on days 1 to 28 in the remaining cycles. For arm C, the once-daily dose or the twice-daily dose with a 12-hour interval was orally administered on day 1 of the run-in period, followed by a 96-hour no-drug period. This was followed by the start of cycle 1 day 1, during which patients received tinengotinib once daily or twice daily consecutively through 28-day treatment cycles.

All participants provided informed consent before the conduct of any protocol-specific assessments. This study was conducted in accordance with the protocol and the ethical principles of the Declaration of Helsinki and Council for International Organizations of Medical Sciences International Ethical Guidelines, applicable International Council for Harmonization and Good Clinical Practice guidelines, and applicable laws and regulations. The protocol and informed consent form were approved by the institutional review board/independent ethics committee before the study was initiated.

### Patients

This study included patients with cholangiocarcinoma, HER2-negative breast cancer (including triple-negative breast cancer), bladder cancer, prostate cancer [including metastatic castration-resistant prostate cancer (mCRPC)], sarcoma, hepatocellular carcinoma, small cell lung cancer, thyroid cancer, gastric cancer, gallbladder cancer, and other advanced solid tumors. Other inclusion criteria included ≥18 years of age, histopathologic or cytologic confirmation of locally advanced or metastatic solid tumors with no available standard therapeutic treatment options, at least 1 measurable lesion as defined by the Response Evaluation Criteria in Solid Tumors version 1.1 (RECIST v1.1) guidelines, and an Eastern Cooperative Oncology Group performance status of 0 or 1. Additionally, adequate organ function (as defined in Supplementary Table S1) was confirmed at screening and within 10 days of treatment initiation.

Exclusion criteria included any hematologic malignancy, including leukemia, lymphoma, and multiple myeloma; a history of primary central nervous system tumors or carcinomatous meningitis; certain mood disorders; impaired cardiac function or significant cardiac diseases; uncontrolled hypertension; impaired gastrointestinal (GI) function; and other concurrent severe and/or uncontrolled concomitant medical conditions. Additionally, patients were excluded if they had received chemotherapy, targeted therapy, or immunotherapy ≤5 half-lives or 3 weeks (6 weeks for nitrosourea or mitomycin C), whichever was shorter, before starting the study drug; wide-field radiotherapy ≤4 weeks or limited-field palliative radiation ≤2 weeks before starting the study drug; or if they had not recovered from side effects of such therapy (see Supplementary Table S1 for full inclusion/exclusion criteria).

### Endpoints

Primary endpoints included safety and tolerability (assessed by Common Terminology Criteria for Adverse Events version 5.0) in arms A and C and PK profiles in arm C. TEAEs were defined as events that initiated after the start of the treatment period or events for which severity worsened after the start of the treatment period. Treatment-related TEAEs were defined as events with suspected relationship with the study treatment. PK assessments included area under the curve (AUC) and maximum observed concentration. Secondary endpoints included preliminary efficacy (per RECIST v1.1) assessed by the objective response rate (ORR), defined as the proportion of patients achieving a complete response (CR) or partial response (PR); progression-free survival (PFS); disease control rate (DCR), defined as CR + PR + stable disease (SD); and duration of response. The clinical benefit rate, defined as CR + PR + SD ≥ 6 months (defined as SD as the best overall response per RECIST v1.1, maintained for a minimum duration of 6 months from the date of first dose until the date of first documented disease progression or death, whichever occurred first), was also assessed. Additional PK endpoints were assessed for arm C, including half-life, time to maximum concentration (t_max_), and volume of distribution. An exploratory biomarker analysis of more than 300 genes was conducted in patients with cholangiocarcinoma, prostate cancer, and breast cancer by tumor type and therapy response using the FoundationOne Liquid CDx assay [Foundation Medicine (RRID: SCR_025628); Supplementary Table S2; ref. 11].

### Statistical analyses

In this early-phase study, we aimed to inform dose selection and signal detection rather than establish definitive efficacy; therefore, analyses were descriptive and hypothesis generating. The Fleming two-stage design allowed efficient identification of promising activity within selected tumor indications while minimizing exposure if activity thresholds were not met. Dose–response and PK relationships were interpreted in an integrated framework to inform the selection of regimens for subsequent evaluation.

A small number of patients (approximately 10–12) were initially planned for enrollment in arm A in the phase Ib part of the study; additional patients were enrolled in phase Ib to ensure that at least 12 patients in the selected indications (cholangiocarcinoma, prostate cancer, and breast cancer) were evaluable for preliminary efficacy analysis before the selection of preferred indications for phase II enrollment. The planned enrollment for arm C was approximately 60 to 68 patients divided into 6 dosage groups. For enrollment in each preferred indication, the Fleming two-stage design was used. The null hypothesis that the true response rate is 5% was tested against a one-sided alternative (Supplementary Table S3). Although the Fleming two-stage design framework was prospectively specified, not all exploratory cohorts ultimately reached the planned stage 1 enrollment target because of evolving program prioritization and transition toward subsequent registrational development.

The safety analysis set included all patients who received ≥1 dose of study treatment. The efficacy analysis set comprised all patients with measurable disease at baseline who received ≥1 dose of study medication and had ≥1 posttreatment tumor assessment (patients in the 12-mg once-daily groups from arms A and C were pooled for efficacy analyses). PK endpoints were assessed among patients in arm C.

Genomic analyses were performed using circulating tumor DNA (ctDNA) profiling data generated using the Foundation Medicine (RRID: SCR_025628) platform. Variant annotation and functional classification were conducted according to the vendor’s standard analytic pipeline, incorporating variant type, predicted functional consequence, ClinVar annotations, and literature-based evidence review. Variant detection was performed using GATK (RRID: SCR_001876).

For mutation association analysis, the Fisher exact test was used to explore associations between genomic alterations and clinical outcomes. Variants observed in fewer than 3 cases were excluded from exploratory biomarker analyses. Exploratory multiple-testing adjustment was performed using the Benjamini–Hochberg method to control the false discovery rate.

For variant allele frequency (VAF) comparisons, paired analyses of longitudinal ctDNA samples from the same patient were conducted using the Wilcoxon matched-pair signed-rank test.

For survival analysis, PFS probabilities were estimated using the Kaplan–Meier method, with group differences assessed using the log-rank test. Hazard ratios and 95% confidence intervals were calculated using univariate Cox proportional hazards models. Similar filtering criteria (variants occurring in <2 cases) and Benjamini–Hochberg correction were applied. Statistical and survival analyses were conducted using R (RRID: SCR_001905) and SAS Statistical Software version 9.4 (RRID: SCR_008567).

For PK analyses, plasma samples from all patients were evaluated for tinengotinib concentration using a validated liquid chromatography–tandem mass spectrometry assay using Analyst TF software (RRID: SCR_015785; values below the lower limit of quantification were reported as 0 ng/mL); missing values were labeled accordingly. Noncompartmental PK analyses were conducted using Phoenix WinNonlin version 8.3.5 or higher (RRID: SCR_017279).

Subgroup analyses were conducted in patients with cholangiocarcinoma, breast cancer, and prostate cancer. Acquired triple-negative breast cancer was defined as prior HR-positive breast cancer with subsequent confirmation of triple-negative status at study enrollment or metastatic recurrence based on available pathology assessment. Given the limited sample size and multiple comparisons inherent to exploratory biomarker analyses, findings should be interpreted cautiously and require prospective validation.

## Results

### Patient disposition and baseline data

Patients were enrolled between July 14, 2021, and March 13, 2023. The study ended on February 20, 2024. In arms A and C, 254 patients with solid tumors were screened and 197 were enrolled and treated (arm A, *n* = 86; arm C, *n* = 111; Supplementary Fig S1). The primary reasons for treatment discontinuation were progressive disease [PD; 116 (58.9%)], adverse events [47 (23.9%)], and withdrawal of consent [15 (7.6%)]. The mean (standard deviation) study duration was 6.5 (5.2) months [median (range), 4.4 (0.4–31) months] in the overall population.

At baseline, the mean (standard deviation) age was 60.1 (11.9) years, and most patients were White [140 (71.1%); Table 1). Most patients had stage IV cancer [180 (91.4%)], and the major primary cancer sites were the colorectum [34 (17.3%)], bile duct [26 (13.2%)], breast [21 (10.7%)], pancreas [21 (10.7%)], and prostate [17 (8.6%)]. Among patients with breast cancer, 7 patients had triple-negative breast cancer and 5 patients had acquired triple-negative breast cancer (i.e., HR-positive status at diagnosis with triple-negative breast cancer at the time of the most recent evaluation) and were included in a hypothesis-generating analysis; no enrolled patients with breast cancer had previously expressed HER2.

**Table 1.** Baseline demographics and disease characteristics.

Characteristic	Arm A	Arm C						Arms A + C	Overall
	12 mg once daily (*n* = 86)	4 mg twice daily (*n* = 18)	6 mg twice daily (*n* = 18)	5 mg once daily (*n* = 14)	8 mg once daily (*n* = 18)	10 mg once daily (*n* = 21)	12 mg once daily (*n* = 22)	12 mg once daily (*n* = 108)	(*N* = 197)
Age, years, mean (s.d.)	59 (12.3)	59.1 (12.5)	58.7 (10.3)	59.8 (13.5)	62.1 (13)	64.2 (9.6)	61.2 (11.1)	59.4 (12)	60.1 (11.9)
Sex, *n* (%)	​	​	​	​	​	​	​	​	​
Male	45 (52.3)	9 (50)	7 (38.9)	5 (35.7)	5 (27.8)	6 (28.6)	9 (40.9)	54 (50)	86 (43.7)
Female	41 (47.7)	9 (50)	11 (61.1)	9 (64.3)	13 (72.2)	15 (71.4)	13 (59.1)	54 (50)	111 (56.3)
Race, *n* (%)	​	​	​	​	​	​	​	​	​
White	56 (65.1)	14 (77.8)	13 (72.2)	10 (71.4)	15 (83.3)	14 (66.7)	18 (81.8)	74 (68.5)	140 (71.1)
African American or Black	14 (16.3)	1 (5.6)	1 (5.6)	2 (14.3)	2 (11.1)	2 (9.5)	2 (9.1)	16 (14.8)	24 (12.2)
American Indian or Alaska Native	1 (1.2)	0	0	0	0	0	0	1 (0.9)	1 (0.5)
Asian	9 (10.5)	1 (5.6)	2 (11.1)	2 (14.3)	0	1 (4.8)	0	9 (8.3)	15 (7.6)
Mixed/other	6 (7)	2 (11.1)	2 (11.1)	0	1 (5.6)	4 (19)	2 (9.1)	8 (7.4)	17 (8.6)
Ethnicity, *n* (%)	​	​	​	​	​	​	​	​	​
Hispanic or Latino	13 (15.1)	3 (16.7)	3 (16.7)	1 (7.1)	2 (11.1)	4 (19)	5 (22.7)	18 (16.7)	31 (15.7)
Non-Hispanic or Latino	71 (82.6)	13 (72.2)	14 (77.8)	13 (92.9)	15 (83.3)	16 (76.2)	17 (77.3)	88 (81.5)	159 (80.7)
Weight, kg, mean (s.d.)	77 (18.4)^a^	72.5 (22.7)	72.8 (20.2)	74 (22.1)	71.2 (19.3)	71 (16)	77.3 (19.6)^a^	77.4 (18.4)^a^	75 (19.1)^a^
Primary cancer site, *n* (%)									
Colorectum	9 (10.5)	4 (22.2)	8 (44.4)	4 (28.6)	4 (22.2)	3 (14.3)	2 (9.1)	11 (10.2)	34 (17.3)
Bile duct	12 (14)	4 (22.2)	3 (16.7)	1 (7.1)	1 (5.6)	2 (9.5)	3 (13.6)	15 (13.9)	26 (13.2)
Breast	11 (12.8)	0	2 (11.1)	0	0	3 (14.3)	5 (22.7)	16 (14.8)	21 (10.7)
Pancreas	7 (8.1)	4 (22.2)	0	3 (21.4)	2 (11.1)	2 (9.5)	3 (13.6)	10 (9.3)	21 (10.7)
Prostate	15 (17.4)	0	0	0	1 (5.6)	1 (4.8)	0	15 (13.9)	17 (8.6)
Other^b^	32 (37.2)	6 (33.3)	5 (27.8)	6 (42.9)	10 (55.6)	10 (47.6)	9 (40.9)	41 (38)	78 (39.6)
Cancer stage, *n* (%)									
I/II	2 (2.3)	0	1 (5.6)	0	0	1 (4.8)	0	2 (1.9)	4 (2)
III	3 (3.5)	2 (11.1)	1 (5.6)	1 (7.1)	1 (5.6)	2 (9.5)	1 (4.5)	4 (3.7)	11 (5.6)
IV	80 (93)	16 (88.9)	16 (88.9)	13 (92.9)	17 (94.4)	18 (85.7)	20 (90.9)	100 (92.6)	180 (91.4)
ECOG performance status, *n* (%)									
0	19 (22.1)	6 (33.3)	4 (22.2)	5 (35.7)	4 (22.2)	2 (9.5)	2 (9.1)	21 (19.4)	42 (21.3)
1	66 (76.7)	12 (66.7)	13 (72.2)	9 (64.3)	14 (77.8)	19 (90.5)	20 (90.9)	86 (79.6)	153 (77.7)
Missing	1 (1.2)	0	1 (5.6)	0	0	0	0	1 (0.9)	2 (1)

Abbreviations: ECOG, Eastern Cooperative Oncology Group; s.d., standard deviation.

a *N* values for weight for arm A 12 mg once daily, arm C 12 mg once daily, arms A + C 12 mg once daily, and overall were 85, 20, 105, and 194, respectively.

b Type and numbers among “Other” (*n* = 78): ovarian, *n* = 15; lung, *n* = 8; uterine, *n* = 7; salivary gland, *n* = 6; esophageal, *n* = 5; sarcoma, *n* = 5; gallbladder, *n* = 4; head and neck, *n* = 4; stomach, *n* = 4; non-biliary liver, *n* = 3; bladder, *n* = 3; thymus, *n* = 2; thyroid, *n* = 2; and other solid tumor types with only 1 patient enrolled per indication.

Baseline characteristics were generally comparable between tinengotinib dosage groups (Table 1). Approximately 8% of patients had 1 prior line of therapy, 17% had 2 prior lines of therapy, and 74% had ≥3 prior lines of therapy; 7 (3.6%) patients had prior FGFR inhibitor treatment (Supplementary Table S4A). Baseline FGFR status is reported in Supplementary Table S4B, and the representativeness of the study participants is presented in Supplementary Table S5.

### Safety

Treatment-related TEAEs and serious treatment-related TEAEs were reported in 160 (81.2%) and 25 (12.7%) patients, respectively, whereas treatment-related TEAEs leading to study drug discontinuation were reported in 17 (8.6%) patients (Table 2).

**Table 2.** Summary of treatment-related TEAEs by dosage group (safety analysis set).

*n* (%)	Arm A	Arm C						Arms A + C	Overall
	12 mg once daily (*n* = 86)	4 mg twice daily (*n* = 18)	6 mg twice daily (*n* = 18)	5 mg once daily (*n* = 14)	8 mg once daily (*n* = 18)	10 mg once daily (*n* = 21)	12 mg once daily (*n* = 22)	12 mg once daily (*N* = 108)	(*N* = 197)
Treatment-related TEAEs	71 (82.6)	14 (77.8)	16 (88.9)	7 (50)	16 (88.9)	18 (85.7)	18 (81.8)	89 (82.4)	160 (81.2)
Treatment-related serious TEAEs	7 (8.1)	2 (11.1)	5 (27.8)	0	2 (11.1)	5 (23.8)	4 (18.2)	11 (10.2)	25 (12.7)
Treatment-related TEAEs by maximum severity									
Grade 1	7 (8.1)	6 (33.3)	3 (16.7)	1 (7.1)	4 (22.2)	3 (14.3)	1 (4.5)	8 (7.4)	25 (12.7)
Grade 2	21 (24.4)	5 (27.8)	3 (16.7)	0	5 (27.8)	4 (19)	7 (31.8)	28 (25.9)	45 (22.8)
Grade 3	43 (50)	3 (16.7)	9 (50)	6 (42.9)	7 (38.9)	9 (42.9)	10 (45.5)	53 (49.1)	87 (44.2)
Grade 4	0	0	1 (5.6)	0	0	2 (9.5)	0	0	3 (1.5)
Treatment-related TEAEs leading to study drug discontinuation	9 (10.5)	0	2 (11.1)	0	1 (5.6)	3 (14.3)	2 (9.1)	11 (10.2)	17 (8.6)
Treatment-related TEAEs leading to death	0	0	0	0	0	0	0	0	0

NOTE: Adverse events were assigned a severity grade using Common Terminology Criteria for Adverse Events version 5.0. Treatment-related TEAEs were events with a relationship to the study drug reported as missing or suspected.

Treatment-related TEAEs tended to occur more often with increased dosages, but there were no obvious trends in dosing schedules. Treatment-related grade 3 and 4 events were reported in 87 (44.2%) and 3 (1.5%) patients, respectively. The 3 patients with treatment-related grade 4 events experienced a total of 5 events, all of which led to treatment discontinuation (increased alanine and aspartate aminotransferase levels in the 6-mg twice-daily group of arm C; large intestine perforation, hypertension, and subarachnoid hemorrhage in the 10-mg once-daily group of arm C).

The most common treatment-related TEAE was hypertension, reported in 87 (44.2%) patients; of these patients, 30 (15.2%) had grade 3 events and 1 (0.5%) had a grade 4 event. Treatment-related hypertension led to treatment discontinuation in 2 patients (a grade 3 event in the 12-mg once-daily group of arm A and a grade 4 event in the 10-mg once-daily group of arm C). Treatment-related GI disorders were reported in 112 (56.9%) patients, of which stomatitis was the most common [61 (31%) patients; 15 (7.6%) had grade 3 events]. Treatment-related skin and subcutaneous tissue disorders were reported in 50 (25.4%) patients, 40 (20.3%) of whom had palmar–plantar erythrodysesthesia syndrome [9 (4.6%) had grade 3 events]. No treatment-related eye toxicities were reported; grade 1 nail discoloration was reported in 1 patient in the 12-mg once-daily group. Hyperphosphatemia was reported in 18 (9.1%) patients (16 with grade 1 events and 2 with grade 2 events); of these patients, 3 (1.5%) had events related to study treatment (2 with grade 1 events and 1 with a grade 2 event).

In the overall safety population, TEAEs were reported in 191 (97%) patients and serious TEAEs in 104 (52.8%) patients (Supplementary Table S6). In general, no trend was observed in the frequency of TEAEs among dosage groups in arms A and C. TEAEs in the combined 12-mg once-daily group of arms A + C (*n* = 108) trended similar to or below the average in the overall population (*n* = 197). The most common TEAEs were hypertension [99 (50.3%)], diarrhea [75 (38.1%)], and stomatitis [68 (34.5%); Supplementary Table S6], which were mostly grades 1 to 2 in severity (Supplementary Table S7).

Eight (4.1%) TEAEs led to death across treatment groups; none were considered treatment related (Table 2; Supplementary Table S6). TEAEs that led to death included 2 cases of respiratory failure (12-mg once-daily group in arm A), 2 cases of sepsis (8-mg once-daily and 12-mg once-daily groups in arm C), 2 cases of PD (both in the 12-mg once-daily group of arm A), and 1 case each of peritonitis (10-mg once-daily group of arm C) and disseminated intravascular coagulation (12-mg once-daily group of arm C).

### Efficacy

**Figure 1.**
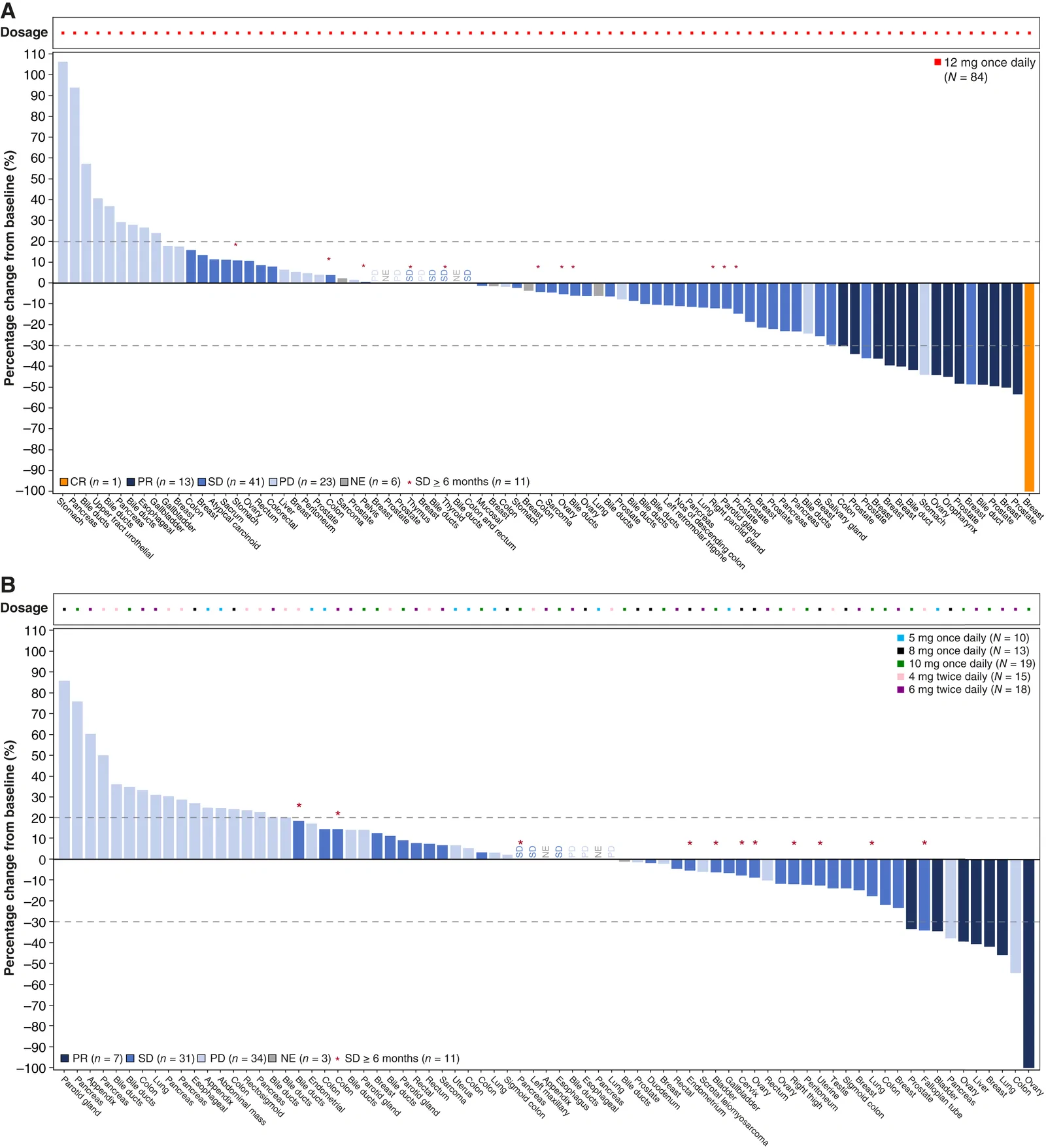
Waterfall plots of the best overall response with tinengotinib in advanced solid tumors according to RECIST v1.1 in the (**A**) combined 12-mg once-daily group in arms A + C and (**B**) other individual dosage groups in arm C. NE, not evaluable.

**Figure 2.**
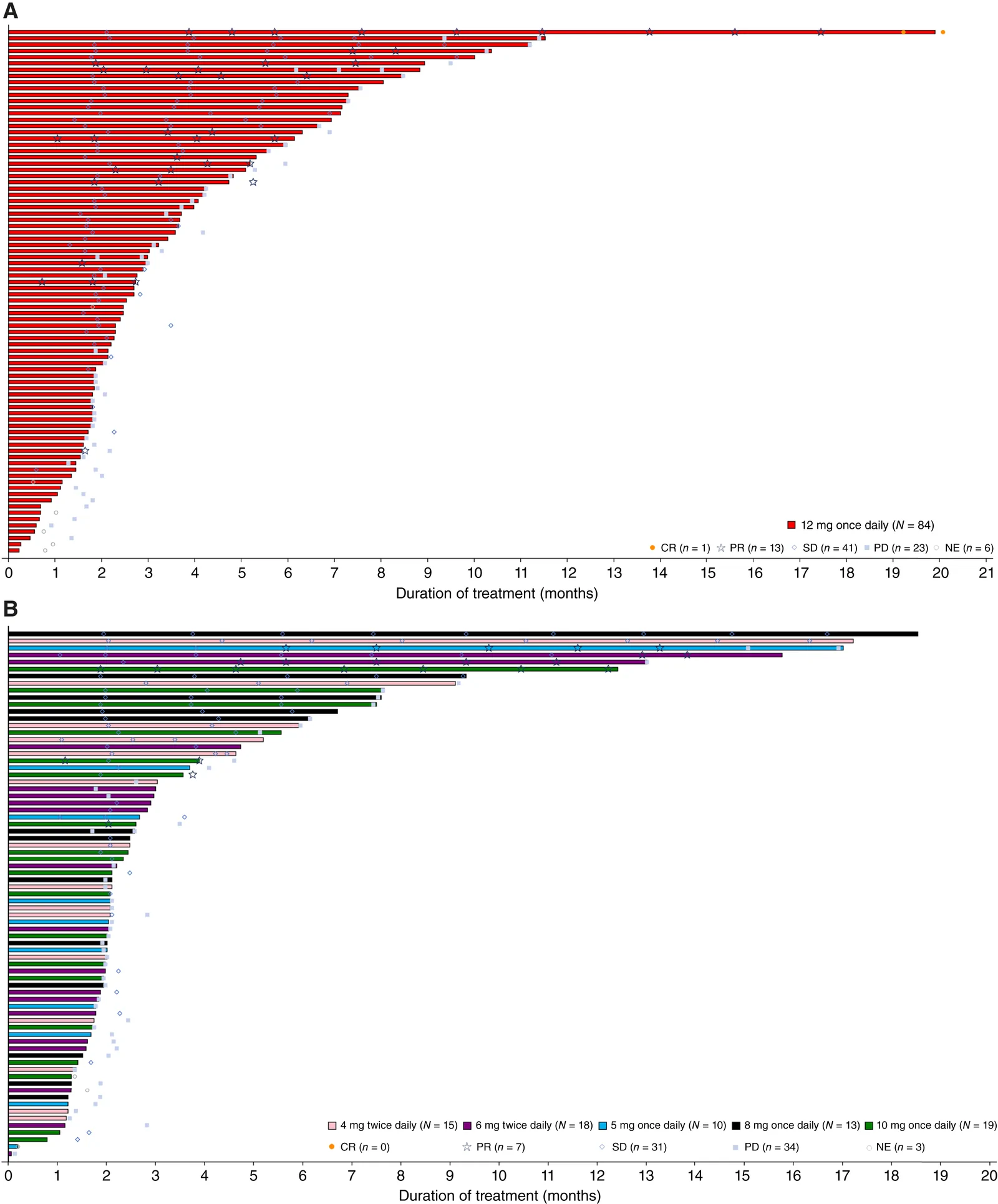
Swimmer plots of the duration and responses of tinengotinib treatment in the (**A**) combined 12-mg once-daily group in arms A + C and (**B**) other individual dosage groups in arm C. NE, not evaluable.

In the overall efficacy population (*n* = 159), CR occurred in 1 (0.6%) and PR in 20 (12.6%) patients (Fig. 1). These included PR in 2 patients at 6 mg twice daily (liver cancer, *n* = 1; lung cancer, *n* = 1), 1 at 5 mg once daily (bladder cancer), 4 at 10 mg once-daily (breast cancer, *n* = 1; prostate cancer, *n* = 1; ovarian cancer, *n* = 2), and 13 at 12 mg once daily (prostate cancer, *n* = 4; breast cancer, *n* = 4; bile ducts cancer, *n* = 2; oropharynx cancer, *n* = 1; ovarian cancer, *n* = 1; colon cancer, *n* = 1). CR occurred in 1 patient at 12 mg once daily (breast cancer; Table 3). The ORR was 13.2% (21/159 patients) in the overall population and trended higher with higher doses. Treatment duration by dosage group is reported in Fig. 2.

**Table 3.** Tumor response assessments per dosage group (efficacy analysis set).

*n* (%)	Arm C					Arms A + C	Overall
	4 mg twice daily (*n* = 15)	6 mg twice daily (*n* = 18)	5 mg once daily (*n* = 10)	8 mg once daily (*n* = 13)	10 mg once daily (*n* = 19)	12 mg once daily (*n* = 84)	(*N* = 159)
ORR	0	2 (11.1)	1 (10)	0	4 (21.1)	14 (16.7)	21 (13.2)
CR	0	0	0	0	0	1 (1.2)	1 (0.6)
PR	0	2 (11.1)	1 (10)	0	4 (21.1)	13 (15.5)	20 (12.6)
SD	7 (46.7)	6 (33.3)	2 (20)	6 (46.2)	10 (52.6)	41 (48.8)	72 (45.3)
SD ≥ 6 months	3 (20.0)	1 (5.6)	0	4 (30.8)	3 (15.8)	11 (13.1)	22 (13.8)
PD	8 (53.3)	9 (50)	6 (60)	7 (53.8)	4 (21.1)	23 (27.4)	57 (35.8)
Not evaluable^a^	0	1 (5.6)	1 (10)	0	1 (5.3)	6 (7.1)	9 (5.7)
DCR^b^ (95% CI)	7 (46.7) [21.3–73.4]	8 (44.4) [21.5–69.2]	3 (30) [6.7–65.2]	6 (46.2) [19.2–74.9]	14 (73.7) [48.8–90.9]	55 (65.5) [54.3–75.5]	93 (58.5) [50.4–66.2]
DOR, events^c^	0	1	1	0	3	11	16
DOR estimate,^d^ months, median (95% CI)	NA (NA–NA)	8.3 (NA–NA)	9.5 (NA–NA)	NA (NA–NA)	2.5 (0.1–NA)	4.2 (1.7–7.7)	4.2 (1.7–8.3)

NOTE: Data are shown as *n* (%) unless otherwise noted.

Abbreviations: CI, confidence interval; DOR, duration of response; NA, not available.

a Had ≥1 post-baseline tumor assessment (efficacy-evaluable) but insufficient data to determine overall response per RECIST 1.1.

b DCR = CR + PR + SD.

c Events included progression or death.

d Calculated using the Kaplan–Meier estimator. 95% CIs were calculated using log–log transformation.

SD occurred in 72 (45.3%) patients, SD ≥ 6 months in 22 (13.8%) patients, and PD in 57 (35.8%) patients in the overall population (Table 3). SD occurred more frequently in the higher dosage groups, whereas PD tended to occur more often in the lower-dosage groups. DCR was 58.5% (93/159 patients) and trended higher with the higher-dosage groups (range, 30%–73.7%). The clinical benefit rate was 27.0%. Similar trends were observed in patients with cholangiocarcinoma, prostate cancer, and breast cancer with ORRs of 13%, 33.3%, and 30%, respectively (Supplementary Table S8). The ORR was 0% among the triple-negative breast cancer (*n* = 7) population versus 60% among the acquired triple-negative breast cancer (*n* = 5) population; DCR was 28.6% versus 100%, respectively (Supplementary Table S9). PFS data are reported in Supplementary Table S10.

### PK profile

During the run-in period in the once-daily groups of arm C, the tinengotinib concentration generally increased with the dose level; however, exposure in the 12-mg group was similar to that in the 10-mg group (Table 4). Similarly, AUC increased with the dose level from 5 to 10 mg, whereas exposure in the 12-mg group was similar to that in the 10-mg group. The t_max_ did not change in different once-daily dosage groups and was approximately 2 to 4 hours. The elimination half-life was similar across all dosage groups at approximately 26 to 34 hours.

**Table 4.** PK parameters of once-daily doses during the run-in period and once-daily and twice-daily doses during cycle 1, day 15 in arm C.

Run-in period: once-daily doses in arm C				
	5 mg once daily (*n* = 14)	8 mg once daily (*n* = 18)	10 mg once daily (*n* = 21)	12 mg once daily (*n* = 22)
C_max_, ng/mL^a^	21.3 (9.7)	28.6 (15.5)	52.1 (22.9)	46.8 (24.4)
t_max_, hours, median (range)^a^	4 (1–24.1)	4.1 (1–8)	2.1 (1–23.7)	4 (1–23.7)
t_1/2_, hours^b^	34.3 (3.5)	26.5 (5)	32.7 (5.2)	28.0 (7.7)
AUC_0–t,_ hours·ng/mL^a^	810.1 (299.7)	1,067.1 (443.6)	1,791.1 (852.5)	1,505.8 (660.6)
CL/F, L/hour^b^	4.5 (0.1)	8.3 (3.8)	5.6 (2.1)	9.0 (4.2)
V_z_/F, L^b^	222.1 (28.2)	318.9 (183)	252.9 (71.5)	340.3 (123.5)

Cycle 1, day 15: once-daily and twice-daily doses in arm C								
​	4 mg twice daily (am; *n* = 18)	4 mg twice daily (pm) (*n* = 18)	6 mg twice daily (am; *n* = 18)	6 mg twice daily (pm; *n* = 18)	5 mg once daily (*n* = 14)	8 mg once daily (*n* = 18)	10 mg once daily (*n* = 21)	12 mg once daily (*n* = 22)
C_max,ss_, ng/mL	49.5 (26.6) *n* = 12	45.9 (26.8) *n* = 8	105.3 (64.5) *n* = 12	99.3 (66) *n* = 8	55.2 (18.9) *n* = 7	89.4 (44.2) *n* = 14	107 (56.2) *n* = 15	110.6 (92.1) *n* = 12
t_max,ss_, hours, median (range)	2.1 (0–11.9) *n* = 12	1.7 (0–11.9) *n* = 8	4 (0.5–11.8) *n* = 12	2 (0–11.8) *n* = 8	2.2 (1–7.8) *n* = 7	3.9 (1.9–23.7) *n* = 14	4 (1–23.5) *n* = 15	2.1 (1–8.3) *n* = 12
t_1/2,ss_, hours	379.7 (809.3) *n* = 5	NA (NA)	61.2 (46.6) *n* = 3	NA (NA)	58.1 (50.2) *n* = 4	31.8 (12.1) *n* = 3	47.5 (27.6) *n* = 4	76.4 (114.8) *n* = 5
AR_AUC_	4.3 (2.5) *n* = 4	4.1 (NA) *n* = 1	NA (NA)	5.3 (NA) *n* = 1	3.2 (1.3) *n* = 5	4.4 (2.4) *n* = 10	2.8 (0.8) *n* = 12	3 (1.7) *n* = 9
AR_Cmax_	3.5 (1.4) *n* = 13	3.2 (1.3) *n* = 8	4.8 (3.4) *n* = 13	4.7 (3.3) *n* = 8	3.2 (2) *n* = 9	3.9 (3.2) *n* = 15	2.4 (0.8) *n* = 15	2.5 (1.1) *n* = 14
AUC_0–t,ss_, hours·ng/mL	514.4 (345.1) *n* = 10	513.7 (318.7) *n* = 7	1,119.1 (769.7) *n* = 13	1,094.2 (688.4) *n* = 8	846 (393.7) *n* = 9	1754.2 (1,032.8) *n* = 14	2,107.5 (1,144.4) *n* = 15	2087.9 (1,694.4) *n* = 12
CL_ss_/F, L/h	9.2 (3.8) *n* = 7	8.6 (1.6) *n* = 3	4.5 (2.6) *n* = 4	9.5 (7.2) *n* = 3	5.4 (2.2) *n* = 5	5.8 (3.6) *n* = 10	6 (2.2) *n* = 12	9.6 (7.8) *n* = 9
V_z,ss_/F, L	3,526.8 (7,271.9) *n* = 5	NA (NA)	275.9 (106.6) *n* = 3	NA (NA)	434.9 (311.3) *n* = 4	397.6 (178.2) *n* = 3	285 (51.9) *n* = 4	624.3 (607.3) *n* = 5

NOTE: Data are shown as mean (standard deviation) unless otherwise noted. Morning (am) and evening (pm) doses are shown for the 4-mg and 6-mg twice-daily groups.

Abbreviations: AR, accumulation ratio; AUC_0–t_, area under the curve from time 0 to the last quantifiable concentration; AUC_0–t,ss_, area under the curve from time 0 to the last quantifiable steady-state concentration; CL/F, apparent clearance; CL_ss_/F, apparent clearance at steady state; C_max_, maximum concentration; C_max,ss_, maximum concentration at steady state; NA, not available; t_1/2_, half-life; t_1/2,ss_, half-life at steady state; t_max,ss_, time to maximum concentration at steady state; V_z_/F, apparent volume of distribution; V_z,ss_/F, apparent volume of distribution at steady state.

a *N* value for the 5-mg once-daily arm was 13.

b *N* values for the 5-mg once-daily, 8-mg once-daily, 10-mg once-daily, and 12-mg once-daily arms were 2, 8, 12, and 11, respectively.

In the multidose analysis, the tinengotinib concentration at day 15 increased with dose (Table 4). Concentrations in the 4-mg twice-daily and 6-mg twice-daily groups were similar to those in the 8-mg and 12-mg once-daily groups, respectively (e.g., AUC exposure was similar at the same total daily dose). Similar to the run-in analysis, t_max_ was approximately 2 to 4 hours. After multiple doses (day 15 analysis), the drug concentration for the same dosage group was higher than the concentration after the first dose during the run-in period (accumulation ratio was ∼2–5; Table 4). Results were generally similar for the day 28 analysis (Supplementary Table S11).

### Exploratory biomarker analysis

In this analysis, patients were classified as nonresponders if they had PD or SD and as responders if they achieved PR. In patients with cholangiocarcinoma (*n* = 18), the most common mutations were *DNMT3A* (61%) and *TP53* (61%; Supplementary Fig S2A). Most *TP53* mutations were found in nonresponders, and most *FGFR2* fusions were in responders (Supplementary Fig S2B). Patients with *EGFR* or *MLL* mutations were associated with shorter median PFS than those without these mutations (1.9 vs. 4.2 months; *P* = 0.0165; 1.9 vs. 2.6 months; *P* = 0.0259, respectively; Supplementary Fig S2C). In patients with prostate cancer (*n* = 10), the most frequently observed alterations included *TP53* (60%), followed by *DNMT3A* (40%) and *TMPRSS2* (40%; Supplementary Fig S3A). Among the genes analyzed, *MYC* alterations were associated with shorter median PFS versus no *MYC* alterations (1.9 vs. 3.6 months; *P* = 0.0289; Supplementary Fig S3B). In patients with breast cancer (*n* = 18), the most common mutations were *TP53* (72%) and *DNMT3A* (50%; Supplementary Fig S4A). *ROS1* mutations were more common (*P* = 0.033) in responders than in nonresponders (Supplementary Fig S4B). There was a reduction in the VAF of 7 patients from baseline to cycle 3 day 1 (*P* < 0.0001), indicating a molecular response with tinengotinib treatment (Supplementary Fig S4C). Furthermore, *ROS1* and *DNMT3A* mutations were higher in patients with acquired triple-negative breast cancer than in those with triple-negative breast cancer (*P* = 0.015 and *P* = 0.0256, respectively; Supplementary Fig S5A). A longer median PFS was observed in patients with acquired triple-negative breast cancer than in those with triple-negative breast cancer (4.3 vs. 2.6 months; *P* = 0.0113; Supplementary Fig S5B).

## Discussion

This multicenter phase Ib/II study demonstrated that tinengotinib exhibited a manageable toxicity profile and produced objective responses across a heterogeneous population of advanced solid tumors in nearly 200 patients. Hypertension represented the most frequent toxicity and was generally manageable with standard antihypertensives and medications and dose modifications (Supplementary Table S12). Importantly, class-associated FGFR toxicities, including ocular and nail toxicities, as well as severe hyperphosphatemia, were infrequent in this study population. Given the multikinase profile of tinengotinib, the observed safety profile may reflect a distinct spectrum of on-target toxicities associated with multikinase inhibition.

Preclinical studies have suggested that tinengotinib may overcome acquired resistance to selective FGFR inhibitors (9, 10). In addition to FGFR inhibition, tinengotinib targets Aurora kinases A/B, VEGFR2, and JAK1/JAK2, providing broader pathway inhibition that may contribute to antitumor activity across multiple tumor types. This multikinase profile may represent a therapeutic advantage in tumors with complex signaling dependencies or adaptive resistance mechanisms.

Clinical outcomes for patients with heavily pretreated solid tumors remain limited across many indications. Late-line therapies in advanced prostate cancer and breast cancer typically demonstrate an ORR of approximately 5% to 20% (12–16). In cholangiocarcinoma, therapeutic options following progression on FGFR inhibitors remain particularly limited, with the reported response rate generally around 5% in later-line systemic therapy settings (17). Within this context, the ORRs observed with tinengotinib in this study (33.3% in prostate cancer, 30% in breast cancer, and 13% in cholangiocarcinoma) suggest encouraging antitumor activity in a heavily pretreated population.

However, the current analysis was exploratory in nature and included heterogeneous populations, including patients with and without FGFR alterations and with variable prior FGFR inhibitor exposure. Therefore, observations with regard to resistance mechanisms and differential FGFR-related toxicity profiles should be interpreted cautiously and considered hypothesis generating.

A trend toward dose-dependent activity was observed, with most responses occurring at the 10- and 12-mg once-daily dose levels. Consistent activity was observed across patients with cholangiocarcinoma, prostate cancer (including mCRPC), and breast cancer (including triple-negative breast cancer), supporting further disease-focused clinical investigation of tinengotinib.

PK analyses showed increasing exposure with the 5- to 12-mg once-daily dose range, with similar exposure observed between the 10- and 12-mg groups. Comparable exposure between once-daily and twice-daily regimens was also observed. Integration of PK, safety, and efficacy findings informed selection of the 10-mg once-daily regimen based on overall therapeutic index considerations.

Exploratory biomarker analyses identified genomic alterations consistent with advanced disease biology across tumor types. In cholangiocarcinoma, *TP53* mutations were detected predominantly in nonresponders, highlighting biological heterogeneity and potential resistance to kinase-directed approaches, whereas *FGFR2* fusions were present among responders, consistent with mechanistic target engagement. The association of *EGFR* and *MLL* alterations with shorter PFS may reflect activation of alternative oncogenic signaling pathways contributing to relative treatment resistance.

In prostate cancer, emerging evidence suggests that *MYC* signaling may facilitate the transition from an androgen receptor–dependent state to a neuroendocrine phenotype through transcriptional and epigenetic reprogramming associated with lineage plasticity (18–20). Consistent with these preclinical findings, our clinical observation that *MYC* alterations were associated with shorter PFS may provide early clinical support for the biological relevance of *MYC*-associated lineage plasticity in treatment resistance.

In breast cancer, *ROS1* alterations were observed more frequently among responders; however, this observation arose from an exploratory analysis involving a limited number of patients and should be interpreted cautiously. No formal multiadjusted biomarker efficacy testing was performed, and the biological significance and pathogenicity of the predominantly nonfusion ROS1 variants remain uncertain. Reductions in ctDNA VAF during treatment further supported pharmacodynamic target engagement and highlighted the potential role of molecular monitoring.

Among patients with acquired triple-negative breast cancer, enrichment of *ROS1* and *DNMT3A* alterations and favorable outcomes relative to primary triple-negative breast cancer raises the possibility that a lineage-switched phenotype may exhibit increased dependence on kinase signaling pathways. Given the limited sample size and differences in prior treatment exposure between groups, these findings should be considered exploratory and hypothesis generating.

Overall, all biomarker analyses were exploratory and hypothesis generating, and no formal multiplicity-adjusted biomarker efficacy testing was performed. Additional limitations include heterogeneous ctDNA fractions, multiple comparisons, uncertain functional relevance of certain alterations, and potential clonal hematopoiesis of indeterminate potential (CHIP)-related confounding in ctDNA-based analyses. These findings require prospective validation in large biomarker-defined cohorts.

The preliminary safety and efficacy findings from this study informed dose selection for ongoing clinical development. Two phase II trials are evaluating a tinengotinib 10-mg once-daily dose in patients with mCRPC progressing on hormone therapy (ClinicalTrials.gov identifier: NCT06457919) and in patients with breast cancer (ClinicalTrials.gov identifier: NCT07498478). In parallel, a phase III randomized trial is assessing tinengotinib in *FGFR2*-altered unresectable or metastatic cholangiocarcinoma following prior chemotherapy and FGFR inhibitor therapy (ClinicalTrials.gov identifier: NCT07328919).

Limitations of this phase Ib/II study included the absence of randomization or a comparator arm, modest cohort sizes within tumor subgroups, and variability in biomarker sampling. Future randomized studies with larger biomarker-stratified populations are needed to further characterize efficacy and predictive biomarkers of response. Furthermore, in slowly progressive tumor types, including hormone-resistant prostate cancer, SD ≥ 6 months may be considered supportive but not definitive evidence of antitumor activity. Nonetheless, the strengths of this study include the large heterogeneous population, evaluation across multiple dose levels and schedules, and integration of PK and translational analyses, providing a strong foundation for subsequent indication-specific development.

In conclusion, tinengotinib, a multikinase inhibitor targeting Aurora kinases A/B, FGFR1 to FGFR3, VEGFR2, and JAK1/JAK2, demonstrated manageable toxicity and antitumor activity in 197 patients with advanced solid tumors. The infrequent occurrence of class-associated FGFR toxicities may reflect a distinct spectrum of on-target toxicities associated with multikinase inhibition. Exploratory biomarker analyses identified potential genomic correlates of responses and resistance, including findings supporting emerging *MYC*-associated lineage plasticity biology in prostate cancer. Overall, this multicenter phase Ib/II study provided integrated PK, safety, efficacy, and translational biomarker data across multiple dose levels and schedules, supporting the continued clinical development of tinengotinib in biomarker-informed studies.

## Supplementary Material

Supplementary Figure S1 Patient disposition.

Supplementary Figure S2 Overview of baseline mutations among patients with cholangiocarcinoma (A), selected biomarkers among responders and nonresponders (B), correlative analyses between PFS and biomarkers (C).

Supplementary Figure S3 Overview of baseline mutations among patients with prostate cancer (A) and correlative analyses between PFS and biomarkers (B).

Supplementary Figure S4 Overview of baseline mutations among patients with breast cancer (A), selected mutations in responders versus nonresponders (B), and relative change in VAF from baseline to cycle 3 days 1 (C).

Supplementary Figure S5 Frequency of selected genomic alterations and PFS in TNBC and aTNBC.

Supplementary Table S1 Inclusion and Exclusion Criteria.

Supplementary Table S2 Gene List for the FoundationOne Liquid CDx Assay.

Supplementary Table S3 Fleming’s Two-Stage Design for Enrollment of Each Preferred Indication.

Supplementary Table S4 Prior Lines of Therapies for Cholangiocarcinoma, Breast Cancer, and Prostate Cancer Populations as well as Total Population (A) and Baseline FGFR Status (B).

Supplementary Table S5 Representativeness of Study Participants.

Supplementary Table S6 Summary of TEAEs by Dosage Group (Safety Analysis Set).

Supplementary Table S7 Most Common TEAEs and Severity by Dosage Group (Safety Analysis Set).

Supplementary Table S8 Tumor Response Assessments per Dosage Group by Tumor Type.

Supplementary Table S9 ORR and DCR among Breast Cancer Population.

Supplementary Table S10 Time to PFS According to RECIST 1.1.

Supplementary Table S11 Pharmacokinetic Parameters of Doses at Cycle 1 Day 28 in Arm C.

Supplementary Table S12 Hypertension Management Guidelines.

## Data Availability

The data generated in this study are not publicly available because they are part of an ongoing clinical development program and may be used to support future regulatory submissions. Data are available upon request to the corresponding author.
