# Supplementary material for: Multikinase Inhibition with Tinengotinib in Advanced Solid Tumors: Results from a Multicenter Phase Ib/II Trial
Source: Clin Cancer Res. 2026 Jul 23;32(21):4874–84. doi: 10.1158/1078-0432.CCR-26-1076 (PMC13725326; doi:10.1158/1078-0432.CCR-26-1076)

Fig S1. Patient disposition.
For all efficacy analyses, the patient population from the 12-mg QD group in Arms A and C was combined. Patients excluded from the efficacy-evaluable population primarily included those without post-baseline tumor assessments, patients who withdrew consent prior to efficacy evaluation, patients lost to follow-up before the first scheduled assessment, and patients with insufficient evaluable data for RECIST-based response determination.
AE, adverse event; BID, twice daily; PD, progressive disease; QD, once daily.

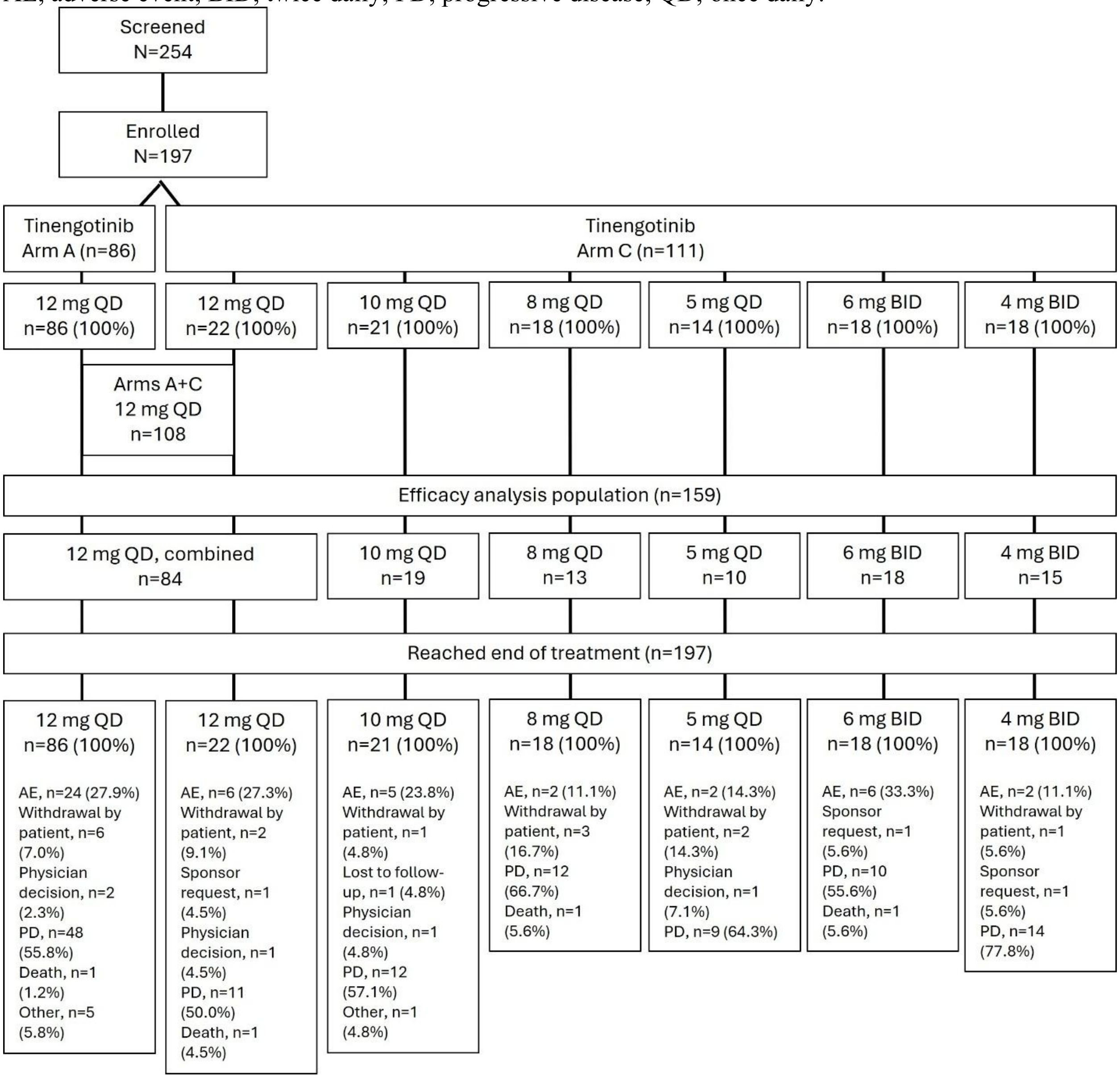

Supplement: Supplementary file 1 [file ccr-26-1076_supplementary_figure_s1_suppfs1.pdf]
