# Supplementary material for: Multikinase Inhibition with Tinengotinib in Advanced Solid Tumors: Results from a Multicenter Phase Ib/II Trial
Source: Clin Cancer Res. 2026 Jul 23;32(21):4874–84. doi: 10.1158/1078-0432.CCR-26-1076 (PMC13725326; doi:10.1158/1078-0432.CCR-26-1076)
Supplement: Supplementary file 2 [file ccr-26-1076_supplementary_figure_s2_suppfs2.pdf]

Fig S2. Overview of baseline mutations among patients with cholangiocarcinoma (A), selected biomarkers among responders and nonresponders (B), correlative analyses between PFS and biomarkers (C).

Panel A: Analysis of liquid biopsy samples includes 18 evaluable CCA patients based on next-generation sequencing (Foundation Medicine, Inc) report. The most common mutations were *DNMT3A* (61.1%), TP53 (61.1%), and KRAS (44.4%).

Panel B: Association of genomic biomarkers with response to tinengotinib in patients with cholangiocarcinoma.

Panel C: Kaplan-Meier plot showing median PFS in C1: patients with (red line) and without (blue line) EGFR mutations, Median PFS was 4.2 months vs 1.9 months in EGFR negative versus EGFR positive patients (*P*=0.0165); C2: patients with (red line) and without (blue line) MLL mutations, Median PFS was 2.6 months vs 1.9 months in MLL negative versus MLL positive patients (*P*=0.0259). Abbreviations: EGFR, endothelial growth factor receptor; PFS, progression-free survival.

A

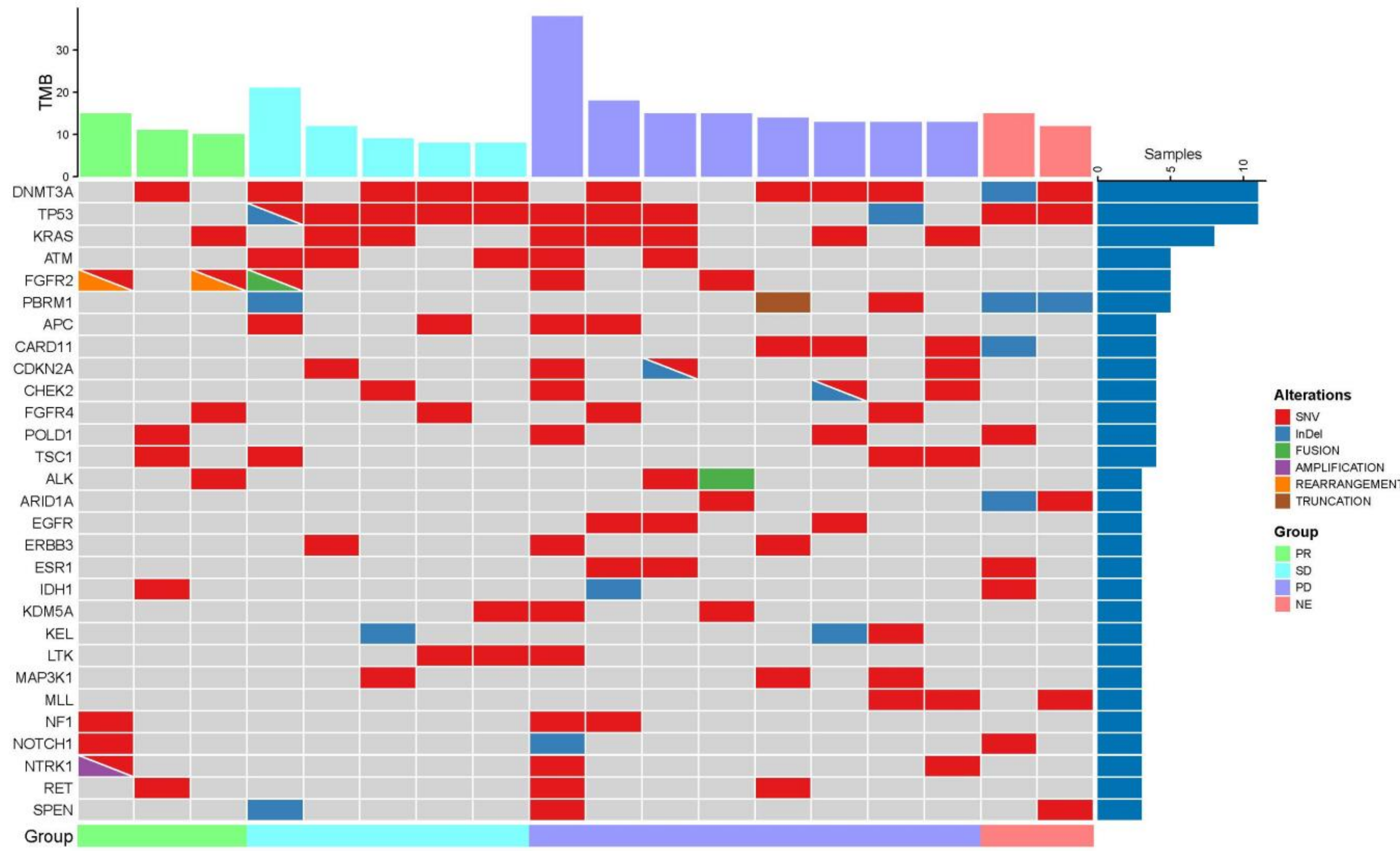

B

Biomarker	Responders (PR)	Nonresponders (SD+PD)	P-value
TP53:mutation positive	0/3 (0%)	9/13 (69%)	0.063
FGFR2:sv positive	2/3 (67%)	1/13 (8%)	0.071
FGFR2:mutation positive	2/3 (67%)	3/13 (23%)	0.214

C1

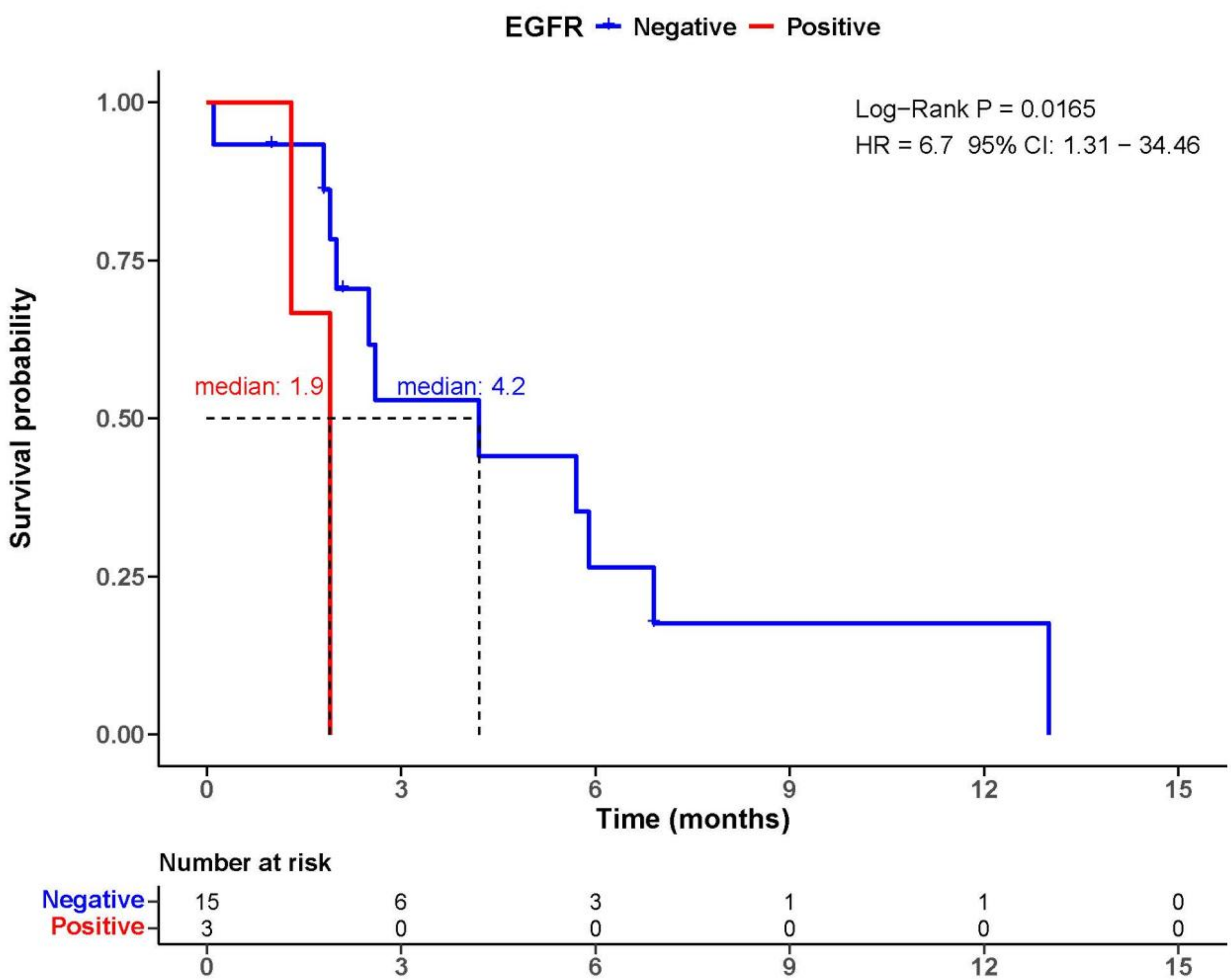

C2

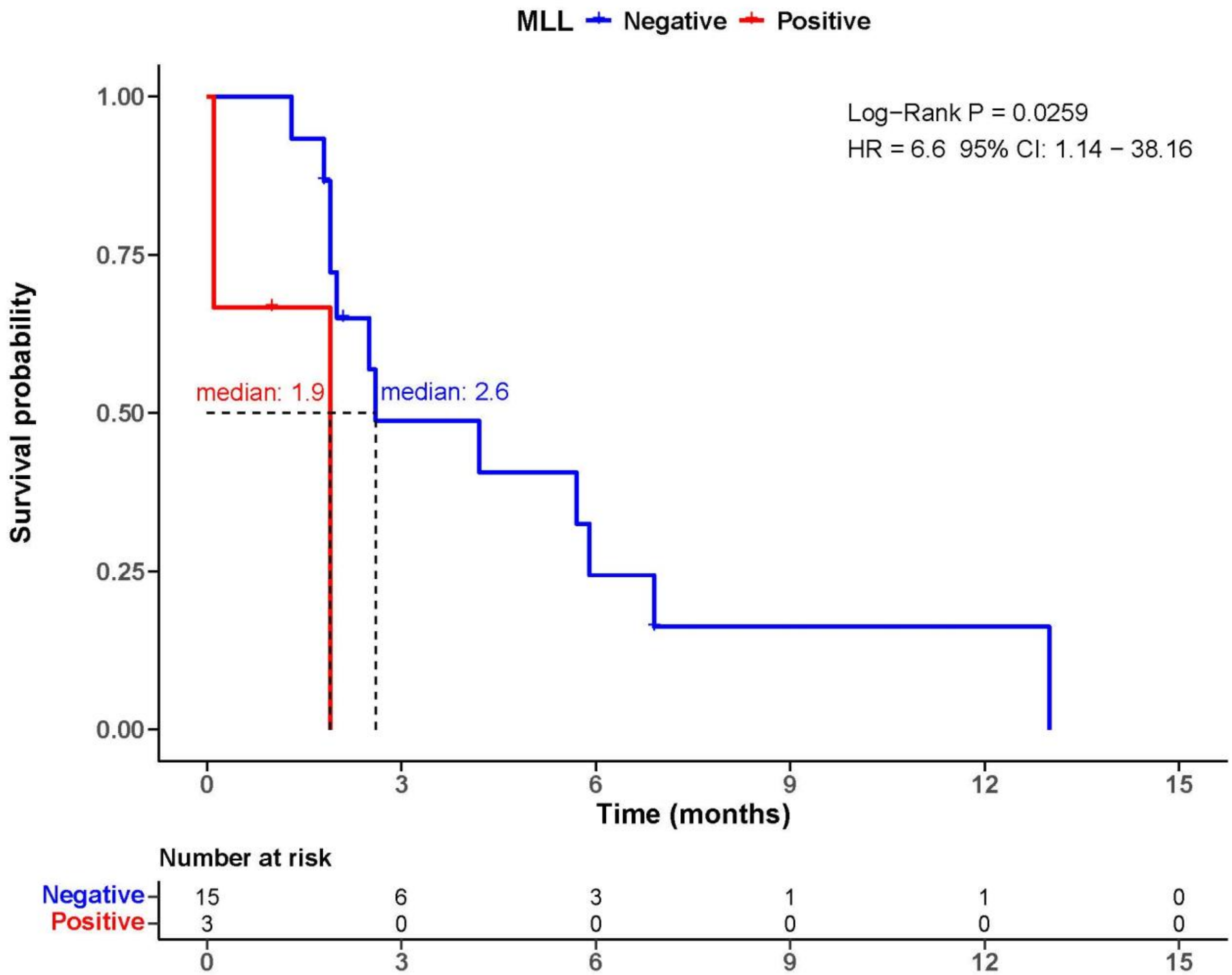

MLL Negative Positive
Log−Rank P = 0.0259
HR = 6.6 95% CI: 1.14 − 38.16
Survival probability
1.00
0.75
0.50
0.25
0.00
median: 1.9
median: 2.6
0
3
6
9
12
15
Time (months)
Number at risk
Negative 15 6 3 1 1 0
Positive 3 0 0 0 0 0
