# Supplementary material for: Multikinase Inhibition with Tinengotinib in Advanced Solid Tumors: Results from a Multicenter Phase Ib/II Trial
Source: Clin Cancer Res. 2026 Jul 23;32(21):4874–84. doi: 10.1158/1078-0432.CCR-26-1076 (PMC13725326; doi:10.1158/1078-0432.CCR-26-1076)

Fig S3. Overview of baseline mutations among patients with prostate cancer (A) and correlative analyses between PFS and biomarkers (B).

Panel A: Analysis of liquid biopsy samples includes 10 evaluable patients with prostate cancer based on next-generation sequencing (Foundation Medicine, Inc) report. The most common mutations were TP53 (60%), AR (40%), DNMT3A (40%), and TMPRSS2 (40%).

Panel B: Kaplan-Meier plot showing median PFS in patients with (red line) and without (blue line) MYC mutations. Median PFS was 3.6 months vs 1.9 months in MYC negative versus MYC positive patients (*P*=0.029). Abbreviations: PD, progressive disease; PFS, progression-free survival; PR, partial response; SD, stable disease.

A

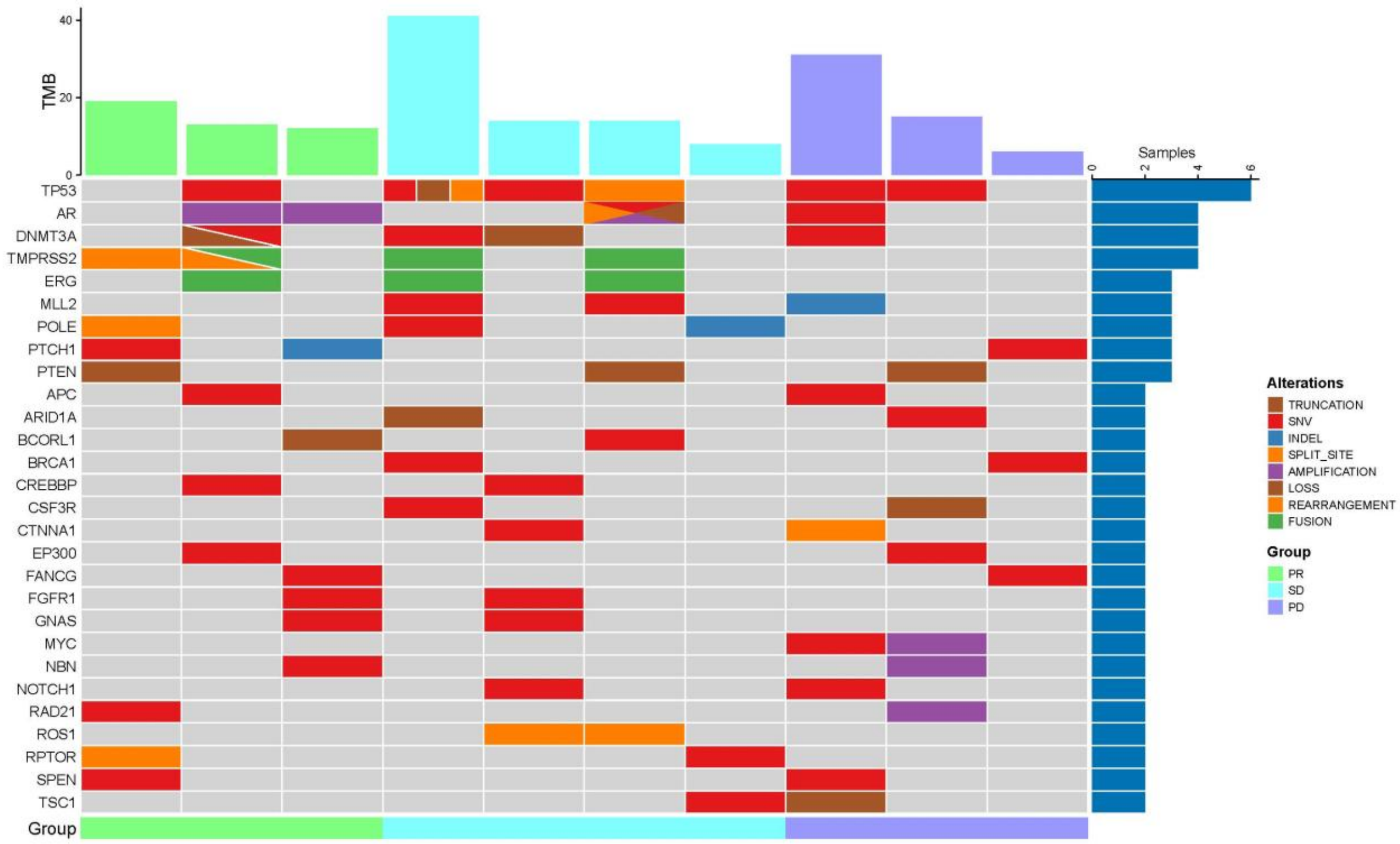

B

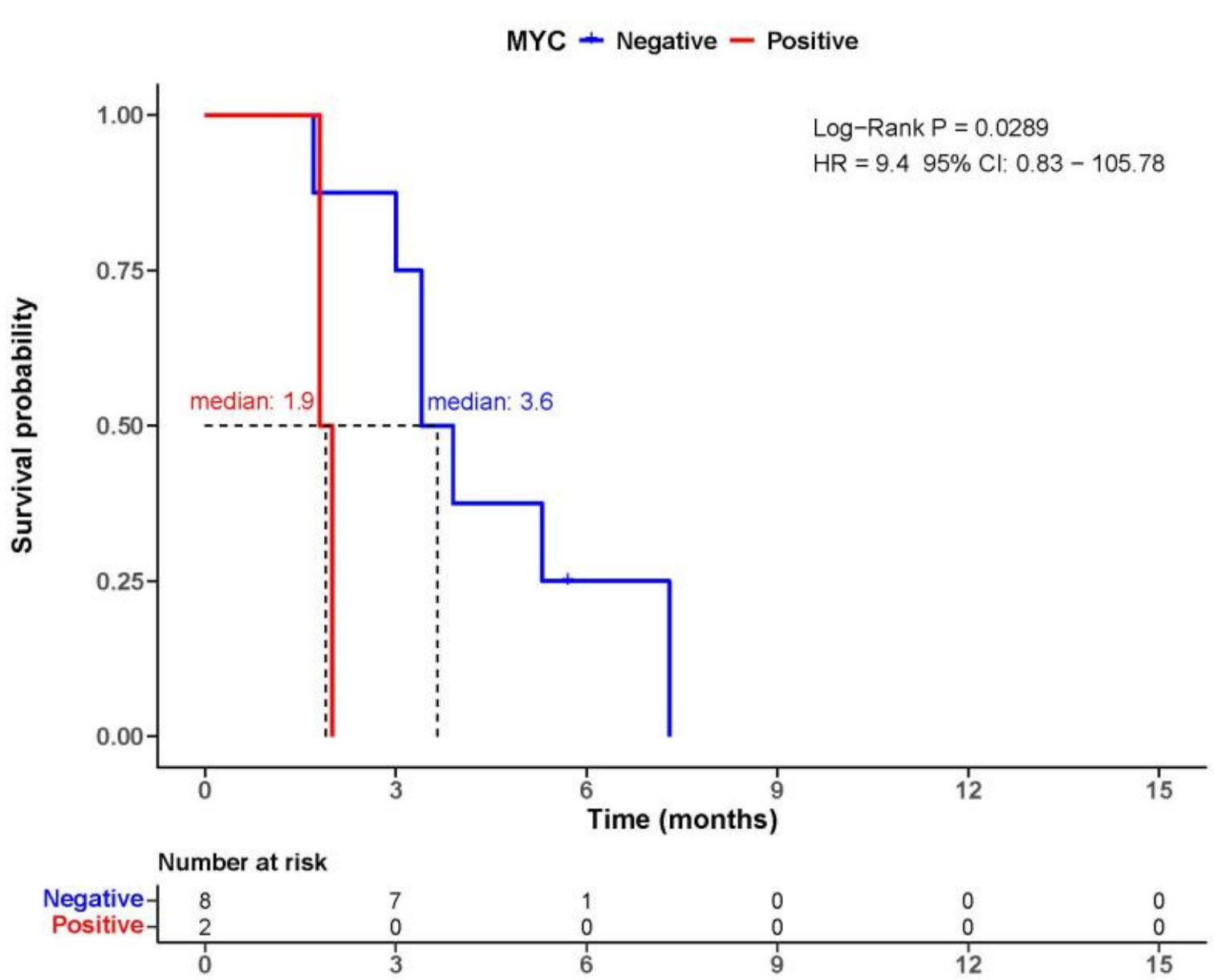

Supplement: Supplementary file 3 [file ccr-26-1076_supplementary_figure_s3_suppfs3.pdf]
