# Supplementary material for: Multikinase Inhibition with Tinengotinib in Advanced Solid Tumors: Results from a Multicenter Phase Ib/II Trial
Source: Clin Cancer Res. 2026 Jul 23;32(21):4874–84. doi: 10.1158/1078-0432.CCR-26-1076 (PMC13725326; doi:10.1158/1078-0432.CCR-26-1076)

Fig S4. Overview of baseline mutations among patients with breast cancer (A), selected mutations in responders versus nonresponders (B), and relative change in VAF from baseline to cycle 3 day 1 (C).

Panel A: Analysis of liquid biopsy samples includes 18 evaluable patients with breast cancer based on next-generation sequencing (Foundation Medicine, Inc) report. *TP53* (72%), *DNMT3A* (50%), *APC* (33%), *PIK3CA* (33%), *FGFR2* (33%), and *PTEN* (33%) were the most common mutations.

Panel B: Association of genomic biomarkers with response to tinengotinib in breast patients.

Panel C: There was a reduction in VAF of 7 patients from baseline to C3D1 (*P*<0.0001), indicating a molecular response with tinengotinib treatment. Abbreviations: C3D1, cycle 3 day 1; NE, not evaluable; PD, progressive disease; PR, partial response; SD, stable disease; VAF, variant allele frequency

A

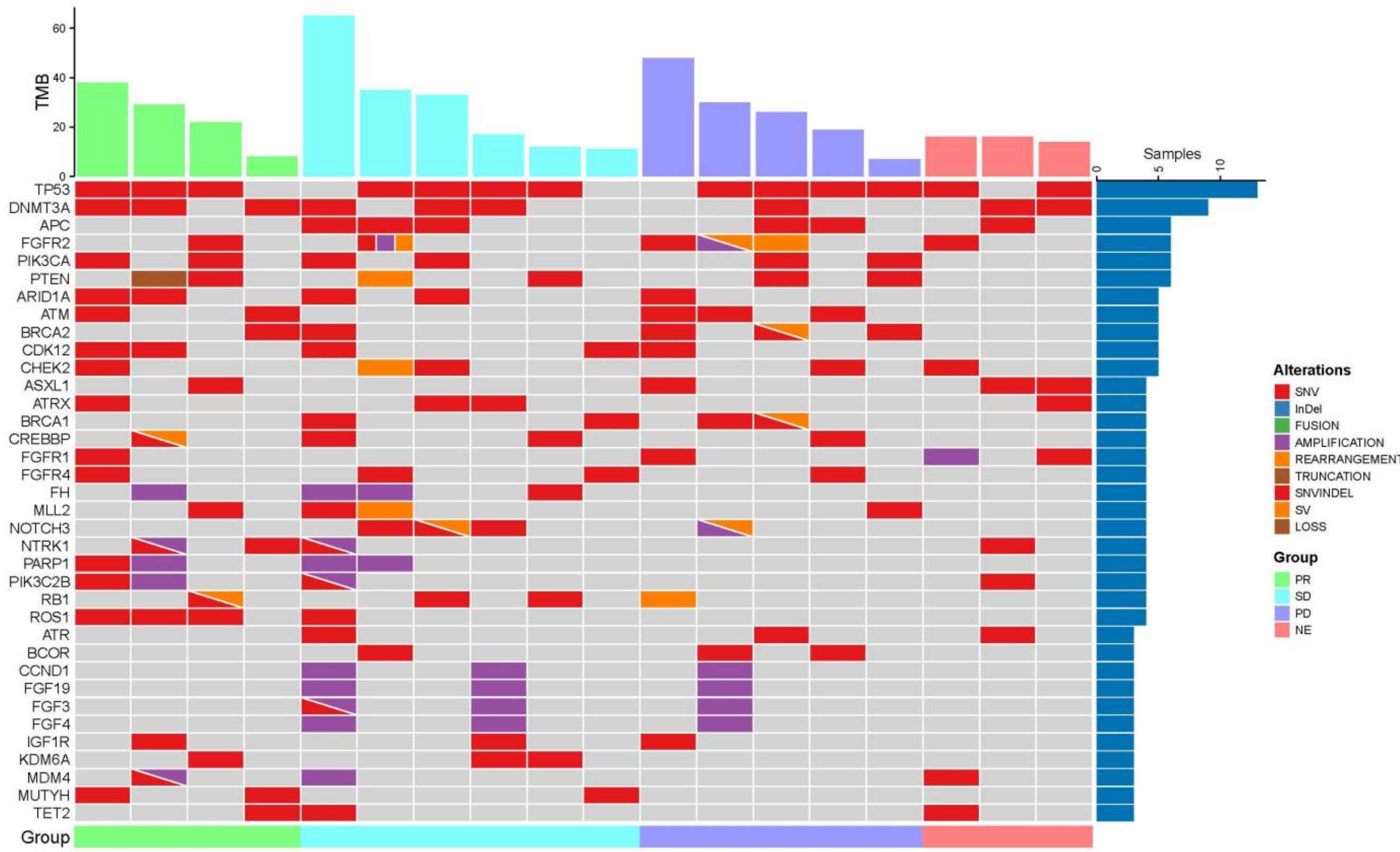

B

Biomarker	Responders (PR)	Non-responders (SD+PD)	*P*-value
ROS1:mutation positive	3/4 (75%)	1/11 (9%)	0.033
MUTYH:mutation positive	2/4 (50%)	1/11 (9%)	0.154
NTRK1:mutation positive	2/4 (50%)	1/11 (9%)	0.154

C

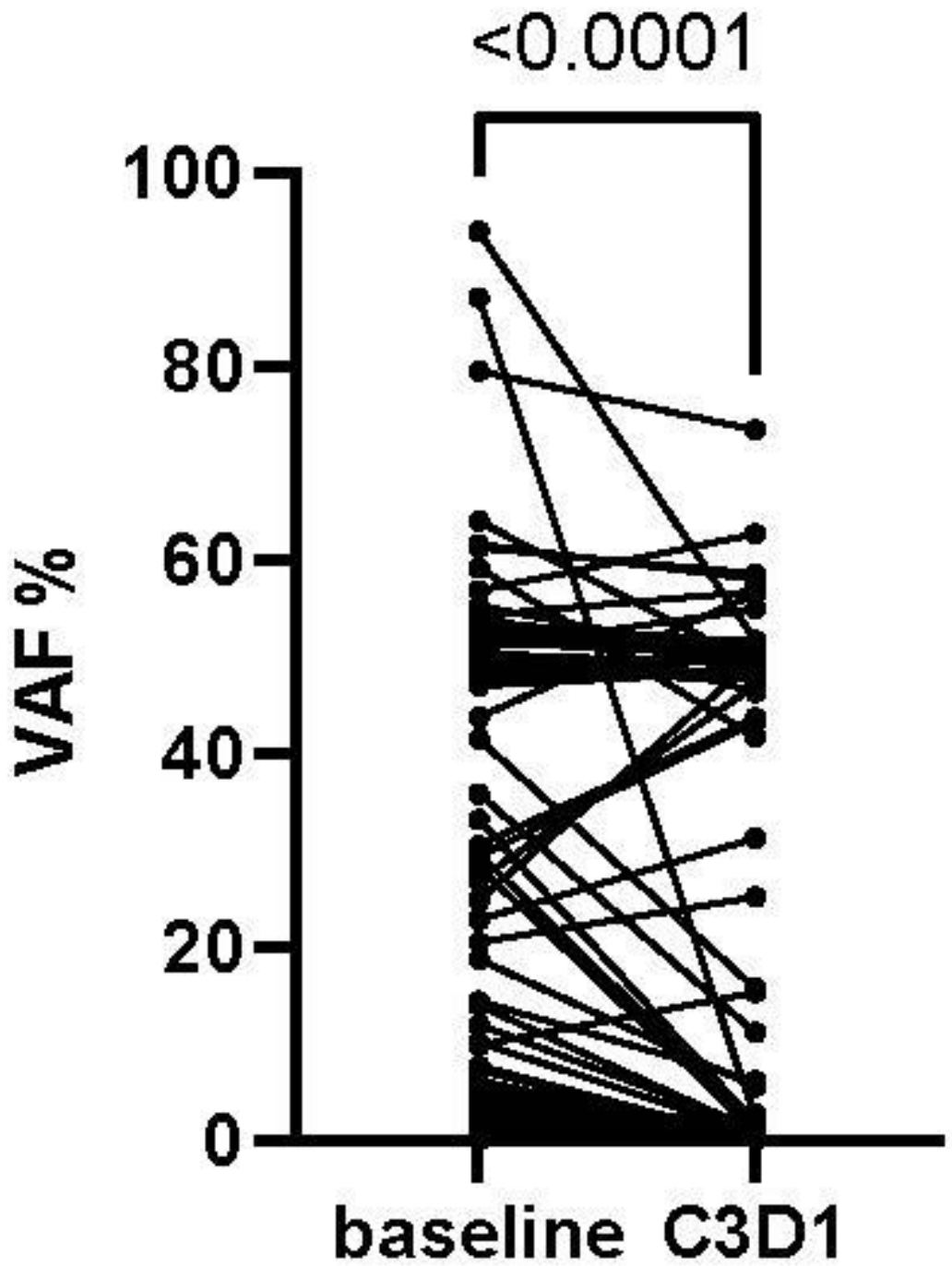

Supplement: Supplementary file 4 [file ccr-26-1076_supplementary_figure_s4_suppfs4.pdf]
