# Supplementary material for: Multikinase Inhibition with Tinengotinib in Advanced Solid Tumors: Results from a Multicenter Phase Ib/II Trial
Source: Clin Cancer Res. 2026 Jul 23;32(21):4874–84. doi: 10.1158/1078-0432.CCR-26-1076 (PMC13725326; doi:10.1158/1078-0432.CCR-26-1076)

Fig S5. Frequency of selected genomic alterations and PFS in TNBC and aTNBC.
Selected genomic alterations in patients with TNBC versus aTNBC (A), PFS comparison between patients with TNBC and aTNBC (B), and FDR correction and q-values on the biomarker analyses (C).
Panel A: Observed enrichment of *ROS1* and *DNMT3A* alterations in aTNBC relative to TNBC. Values represent the number and percentage of alteration-positive cases within each subgroup. *P* values reflect exploratory between-group comparisons.
Panel B: Kaplan-Meier estimates of median PFS in TNBC (blue line) and aTNBC (red line). Median PFS was 2.6 months and 4.3 months for TNBC and aTNBC, respectively (*P*=0.0113).
Abbreviations: aTNBC, acquired TNBC; BH, Benjamini–Hochberg; FDR, false discovery rate; PFS, progression-free survival; TNBC, triple-negative breast cancer

A

Biomarker	TNBC	aTNBC	*P*-value
ROS1:mutation positive	0/8 (0%)	4/6 (67%)	0.015
DNMT3A:mutation positive	1/8 (13%)	5/6 (83%)	0.026

B

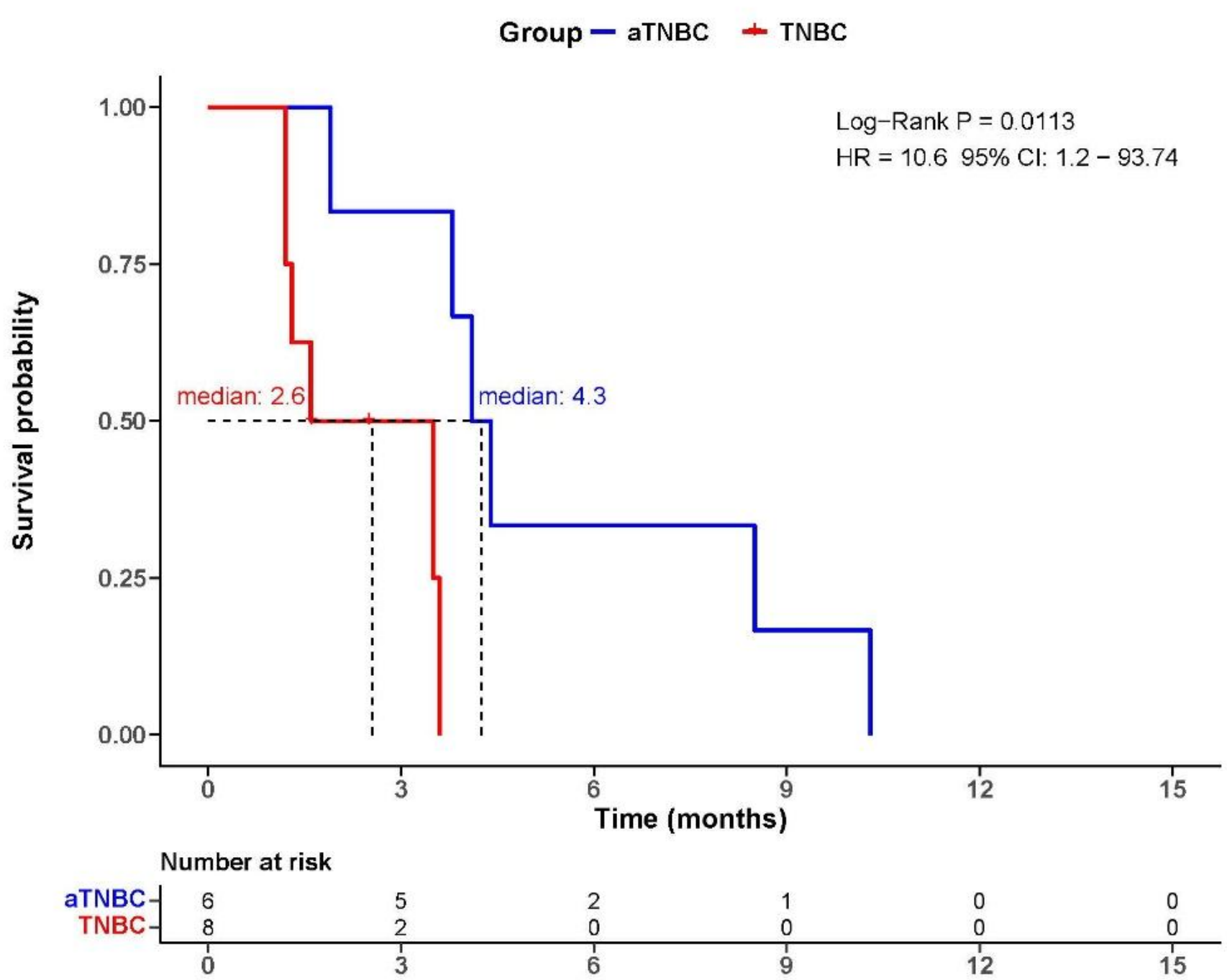

Supplement: Supplementary file 5 [file ccr-26-1076_supplementary_figure_s5_suppfs5.pdf]
