# Supplementary material for: Multikinase Inhibition with Tinengotinib in Advanced Solid Tumors: Results from a Multicenter Phase Ib/II Trial
Source: Clin Cancer Res. 2026 Jul 23;32(21):4874–84. doi: 10.1158/1078-0432.CCR-26-1076 (PMC13725326; doi:10.1158/1078-0432.CCR-26-1076)
Supplement: Supplementary file 6 [file ccr-26-1076_supplementary_table_s10_suppts10.pdf]

Supplementary Table S10. Time to PFS According to RECIST 1.1

PFS	12 mg QD (n=67)	4 mg BID (n=15)	6 mg BID (n=18)	5 mg QD (n=10)	8 mg QD (n=13)	10 mg QD (n=19)	12 mg QD (n=17)	12 mg QD (A+C) (n=84)	Total (N=159)
PFS events, n (%)	53 (79.1%)	12 (80.0%)	14 (77.8%)	9 (90.0%)	10 (76.9%)	13 (68.4%)	11 (64.7%)	64 (76.2%)	122 (76.7%)
Death is first event	12 (17.9%)	1 (6.7%)	4 (22.2%)	1 (10.0%)	1 (7.7%)	4 (21.1%)	2 (11.8%)	14 (16.7%)	25 (15.7%)
Progression is first event	41 (61.2%)	11 (73.3%)	10 (55.6%)	8 (80.0%)	9 (69.2%)	9 (47.4%)	9 (52.9%)	50 (59.5%)	97 (61.0%)
Censored subjects, n (%)	14 (20.9%)	3 (20.0%)	4 (22.2%)	1 (10.0%)	3 (23.1%)	6 (31.6%)	6 (35.3%)	20 (23.8%)	37 (23.3%)
No documented progression and no death	14 (20.9%)	3 (20.0%)	4 (22.2%)	1 (10.0%)	3 (23.1%)	6 (31.6%)	6 (35.3%)	20 (23.8%)	37 (23.3%)
PFS estimate (months) (95% CI)[a]									
75th percentile	6.83 (5.13, 8.44)	7.46 (2.43, NA)	5.95 (2.69, NA)	4.07 (2.10, NA)	7.52 (1.97, NA)	7.43 (4.60, NA)	8.48 (4.24, NA)	7.20 (5.95, 9.36)	7.29 (5.95, 8.48)
Median	3.71 (2.07, 5.13)	2.60 (1.38, 7.46)	2.69 (2.04, 5.95)	2.10 (0.756, 4.07)	2.04 (1.87, 7.52)	4.60 (1.97, 7.43)	6.18 (3.12, 8.48)	4.17 (3.29, 5.29)	3.61 (2.60, 4.21)
25th percentile	1.87 (1.68, 2.07)	1.97 (1.25, 2.43)	2.07 (0.131, 2.20)	1.77 (0.756, 2.10)	1.91 (1.71, 2.04)	2.04 (1.74, 4.60)	3.61 (1.91, 4.24)	1.91 (1.81, 2.53)	1.94 (1.87, 2.07)
Kaplan-Meier estimates[b]									
3 months	57.0 (43.5, 68.4)	37.0 (13.8, 60.8)	36.8 (14.3, 59.8)	30.0 (7.11, 57.8)	38.5 (14.1, 62.8)	69.3 (41.2, 86.0)	87.4 (58.1, 96.7)	63.5 (51.7, 73.2)	54.1 (45.6, 61.9)
6 months	28.8 (17.5, 41.1)	27.8 (7.70, 52.7)	22.1 (5.59, 45.3)	15.0 (1.01, 45.7)	38.5 (14.1, 62.8)	30.8 (9.82, 55.0)	51.0 (23.6, 73.0)	33.5 (22.6, 44.7)	30.9 (23.1, 38.9)
12 months	5.5 (1.13, 15.5)	9.3 (0.588, 33.2)	14.7 (2.46, 37.1)	15.0 (1.01, 45.7)	19.2 (3.31, 45.0)	7.7 (0.485, 29.1)	10.6 (0.657, 36.8)	6.8 (1.97, 15.9)	9.8 (5.05, 16.3)

PFS (months) = (1 + date of PFS event or censor – date of first dose) / (365.25 / 12).
[a]Calculated using the Kaplan-Meier estimator. 95% CIs are calculated using the log-log transformation methodology.
[b]Survival rate [n=number of patients at risk, number of cumulative events], 95% CIs are calculated using the log-log transformation methodology
Abbreviations: BID, once daily; CI, confidence interval; QD, once daily; PFS, progression-free survival.
