# Supplementary material for: Multikinase Inhibition with Tinengotinib in Advanced Solid Tumors: Results from a Multicenter Phase Ib/II Trial
Source: Clin Cancer Res. 2026 Jul 23;32(21):4874–84. doi: 10.1158/1078-0432.CCR-26-1076 (PMC13725326; doi:10.1158/1078-0432.CCR-26-1076)
Supplement: Supplementary file 7 [file ccr-26-1076_supplementary_table_s11_suppts11.pdf]

Supplementary Table S11. Pharmacokinetic Parameters of Doses at Cycle 1 Day 28 in Arm C

	**4 mg BID (AM) (n=18)**	**4 mg BID (PM) (n=18)**	**6 mg BID (AM) (n=18)**	**6 mg BID (PM) (n=18)**	**5 mg QD (n=14)**	**8 mg QD (n=18)**	**10 mg QD (n=21)**	**12 mg QD (n=22)**
$C_{max,ss}$, ng/mL	47.9 (23.8) n=12	34.4 (12.3) n=6	102.5 (52.9) n=8	69.0 (52.0) n=6	65.2 (29.0) n=4	69.7 (29.5) n=9	97.8 (28.6) n=7	100.6 (46.3) n=5
$t_{max,ss}$, h, median (range)	4.0 (2.0–12.0) n=12	1.5 (0.0–1.7) n=6	4.0 (1.9–7.9) n=8	1.0 (0.0–12.0) n=6	3.0 (2.1–7.9) n=4	4.0 (1.0–7.9) n=9	2.0 (1.0–8.0) n=7	4.0 (2.1–23.8) n=5
$t_{1/2,ss}$, h	NA (NA)	48235.4 (NA) n=1	NA (NA)	15.6 (NA) n=1	40.4 (29.0) n=2	48.3 (44.3) n=3	31.3 (15.2) n=5	39.7 (NA) n=1
AR_{AUC}	4.8 (NA) n=1	3.1 (1.7) n=3	6.6 (NA) n=1	4.4 (2.5) n=2	5.7 (6.2) n=4	4.4 (3.9) n=7	2.8 (0.9) n=6	2.9 (0.7) n=2
AR_{Cmax}	3.6 (1.6) n=12	2.5 (0.9) n=6	4.3 (1.7) n=8	3.3 (1.4) n=6	5.2 (5.6) n=5	3.7 (3.3) n=9	2.3 (0.8) n=8	2.6 (0.7) n=6
$AUC_{0–t,ss}$, h·ng/mL	397.1 (147.1) n=11	385.7 (111.3) n=6	1018.8 (616.4) n=7	821.2 (622.4) n=5	1131.6 (672.6) n=5	1175.6 (512.9) n=9	1421.6 (586.9) n=8	1621.2 (670.8) n=6
CL_{ss}/F, L/h	8.9 (NA) n=1	12.5 (6.0) n=5	3.0 (NA) n=1	11.1 (5.2) n=5	4.8 (2.4) n=4	7.3 (3.1) n=7	5.9 (1.4) n=6	9.0 (1.4) n=2
$V_{z,ss}/F$, L	NA (NA)	645265.8 (NA) n=1	NA (NA)	242.2 (NA) n=1	212.4 (22.2) n=2	403.9 (140.5) n=3	236.9 (87.6) n=5	573.0 (NA) n=1

Note: Data are shown as mean (standard deviation) unless otherwise noted. Morning (AM) and evening (PM) doses are shown for the 4-mg and 6-mg BID groups.

Abbreviations: AR, accumulation ratio; AUC, area under the curve; $AUC_{0–t,ss}$, area under the curve from time 0 to the last quantifiable steady-state concentration; BID, twice daily; CL_{ss}/F, apparent clearance at steady state; $C_{max,ss}$, maximum concentration at steady state; NA, not available; QD, once daily; $t_{1/2,ss}$, half-life at steady state; $t_{max,ss}$, time to maximum concentration at steady state; $V_{z,ss}/F$, apparent volume of distribution at steady state.
