# Supplementary material for: Multikinase Inhibition with Tinengotinib in Advanced Solid Tumors: Results from a Multicenter Phase Ib/II Trial
Source: Clin Cancer Res. 2026 Jul 23;32(21):4874–84. doi: 10.1158/1078-0432.CCR-26-1076 (PMC13725326; doi:10.1158/1078-0432.CCR-26-1076)
Supplement: Supplementary file 8 [file ccr-26-1076_supplementary_table_s12_suppts12.pdf]

Supplementary Table S12. Hypertension Management Guidelines

Criteria	Action Taken
CTCAE Grade 2 Hypertension<br>SBP ≥140 mmHg or DBP ≥90 mmHg	Continue tinengotinib, monitor blood pressure daily, and start antihypertensive therapy immediately.<br>• For patient without prior hypertension medical history, start one class of antihypertensive drug;<br>• For patient who is taking antihypertensive drug (s), increase the dose of antihypertensive drug(s), or add a different class of antihypertensive drug.<br>• For complicated hypertension, adding ACEI/ARB class antihypertensive drugs is highly preferred subject
CTCAE Grade 3 Hypertension (confirmed via 2 consecutive measurements)<br>SBP ≥160 mmHg or DBP ≥100 mmHg	Continue tinengotinib, monitor blood pressure daily, visit clinic every 2 weeks or more frequently as clinically indicated until BP ≤150/95 mmHg<br>• For patient without prior hypertension medical history, start one class of antihypertensive drug<br>• For patient who is taking antihypertensive drug (s), increase the dose of antihypertensive drug(s), or add a different class of antihypertensive drug<br>• For complicated hypertension, adding ACEI/ARB class antihypertensive drugs is highly preferred
Persistent CTCAE Grade 3 Hypertension<br>SBP ≥160 mmHg or DBP ≥100 mmHg	1. Interrupt tinengotinib until resolved to ≤150/95 mmHg for at least 48 hours, then resume tinengotinib at a lower dose level<br>2. If Grade 3 hypertension recurs on the current dose level, despite optimal antihypertensive therapy, tinengotinib should be interrupted until BP ≤150/95 mmHg for at least 48 hours, then restarted at the next lower dose level<br>3. After 2 dose reductions, any additional dose reduction should be discussed with the Sponsor
CTCAE Grade 4 Hypertension	Discontinue tinengotinib, institute appropriate medical management, and follow up for safety as per protocol.
