# Supplementary material for: Multikinase Inhibition with Tinengotinib in Advanced Solid Tumors: Results from a Multicenter Phase Ib/II Trial
Source: Clin Cancer Res. 2026 Jul 23;32(21):4874–84. doi: 10.1158/1078-0432.CCR-26-1076 (PMC13725326; doi:10.1158/1078-0432.CCR-26-1076)
Supplement: Supplementary file 9 [file ccr-26-1076_supplementary_table_s1_suppts1.pdf]

Supplementary Table S1. Inclusion and Exclusion Criteria

Inclusion Criteria

Patients were eligible to be included in the study only if all the following criteria applied:

1. ≥18 years of age at the time of signing an informed consent
2. Histopathological or cytologically documented locally advanced or metastatic solid tumors that had no available standard therapeutic treatment options
   - **Arm A and Arm C:** Patients with preferred indications such as cholangiocarcinoma, metastatic HER2-negative breast cancer including TNBC, prostate cancer, sarcoma, hepatocellular carcinoma, bladder cancer, small cell lung cancer, thyroid cancer, gastric cancer, gallbladder cancer, and other advanced solid tumors
     Note**:** Patients with documented records of confirmed *FGFR2* alterations and/or HER-negative test results are highly preferred for enrollment.
   - **Arm B:** Patients with metastatic HER2-negative breast cancer including TNBC that failed to respond to standard-of-care treatments
3. At least one measurable lesion as defined by RECIST version 1.1
4. ECOG performance status of 0 or 1
5. Adequate organ function confirmed at screening and within 10 days of initiating treatment, as evidenced by:
   - Absolute neutrophil count $\geq 1.5 \times 10^9$/L
   - Hemoglobin ≥8 g/dL
   - Platelets $\geq 75 \times 10^9$/L
   - AST and ALT ≤2.5 × ULN or ≤5.0 × ULN if liver metastases are present
   - Total bilirubin ≤1.5 × ULN
   - Serum creatinine ≤1.5 × ULN or calculated creatinine clearance ≥50 mL/min as calculated using the Cockcroft-Gault formula
   - Adequate blood coagulation function as evidenced by INR ≤1.5
6. Negative serum pregnancy test result within 72 hours before starting study treatment in all premenopausal women and women <12 months after the onset of menopause
7. Must agree to take sufficient contraceptive methods and to avoid pregnancy during the study and until at least 6 months after ceasing study treatment
8. Able to sign an informed consent and comply with the protocol

Exclusion Criteria

Patients were excluded from the study if any of the following criteria applied:

1. Women who are pregnant or lactating
2. Women of childbearing potential or men with female partners of childbearing potential who do not use adequate birth control
3. Patients with any hematologic malignancy, including leukemia (any form), lymphoma, and multiple myeloma
4. Patients with a history of primary central nervous system tumors or carcinomatous meningitis (also known as leptomeningeal disease)

   ***Note*:** Patients with treated brain metastases who are off corticosteroids and have been clinically stable for 28 days are eligible for enrollment.
5. Patients with the following mood disorders as judged by the investigator or a psychiatrist:
   - Medically documented history of or active major depressive episode, bipolar disorder (I or II), obsessive-compulsive disorder, or schizophrenia; a history of suicidal attempt or ideation, or homicidal ideation (immediate risk of doing harm to others)
   - CTCAE grade ≥3 anxiety

     ***Note*:** Patients with a stable or resolved history of mental illness may be eligible at the investigator's discretion.
6. Impaired cardiac function or significant diseases, including but not limited to any of the following:
   - LVEF <45% as determined by multigated acquisition scan or echocardiogram
   - Congenital long QT syndrome
   - QTcF ≥480 msec on screening electrocardiogram
   - Unstable angina pectoris ≤3 months before starting the study drug
   - Acute myocardial infarction ≤3 months before starting the study drug
7. Patients with uncontrolled hypertension (defined as blood pressure of ≥150 mmHg systolic and/or ≥90 mmHg diastolic at screening)
8. Patients with:
   - Unresolved diarrhea of CTCAE grade ≥2, or
   - Impairment of gastrointestinal function, or
   - Gastrointestinal disease that may significantly alter the absorption of tinengotinib (eg, ulcerative diseases, uncontrolled nausea, vomiting, diarrhea, malabsorption syndrome, or small bowel resection)
9. Other concurrent severe and/or uncontrolled concomitant medical conditions (eg, uncontrolled hypertension, uncontrolled hypertriglyceridemia [triglycerides >500 mg/dL], or active or uncontrolled infection) that could cause unacceptable safety risks or compromise compliance with the protocol
10. Patients who have received chemotherapy, targeted therapy, or immunotherapy ≤5 half-lives or 3 weeks (6 weeks for nitrosourea or mitomycin-C), whichever is shorter, before starting the study drug or who have not recovered from side effects of such therapy
11. Patients who have received wide-field radiotherapy ≤4 weeks or limited-field radiation for palliation ≤2 weeks before starting the study drug or who have not recovered from side effects of such therapy

12. Patients who have undergone major surgery ≤4 weeks before starting the study drug or who have not recovered from the side effects of such therapy.
13. Patients with a bleeding disorder or thrombotic disorder requiring anticoagulant therapy, such as warfarin sodium (Coumadin®) or any other coumarin-derivative anticoagulants (treatment with low molecular weight heparin is allowed).
14. Patients who are currently receiving treatment with strong CYP3A inhibitors or inducers ≤2 weeks before starting the study drug.
15. Known diagnosis of HIV infection (HIV testing is not mandatory; patients with well controlled HIV might be enrolled per the investigator's discretion).
16. Known history of active infection with hepatitis B (eg, HBsAg reactive) or hepatitis C (eg, HCV RNA [qualitative] is detected)
17. Patients who have received a live virus vaccination within 30 days of the planned first dose

    ***Note*:** Seasonal flu and COVID-19 vaccines are permitted.
18. Patients with an inability to swallow or tolerate oral medication
19. Patients who have a history or current evidence of any condition, therapy, or laboratory abnormality that, in the opinion of the investigator, might confound the results of the trial or interfere with the patient's safe participation and compliance in the trial.

Abbreviations: ALT, alanine aminotransferase; AST, aspartate aminotransferase; COVID-19, coronavirus disease 2019; CTCAE, Common Terminology Criteria for Adverse Events; CYP, cytochrome P450; ECOG, Eastern Cooperative Oncology Group; FGFR2, fibroblast growth factor receptor 2; HBsAg, hepatitis B surface antigen; HCV, hepatitis C virus; HER2, human epidermal growth factor receptor 2; HIV, human immunodeficiency virus; INR, international normalized ratio; LVEF, left ventricular ejection fraction; QTcF, corrected QT interval using the Fridericia formula; RECIST, Response Evaluation Criteria in Solid Tumors; TNBC, triple-negative breast cancer; ULN, upper limit of normal.
