# Supplementary material for: Multikinase Inhibition with Tinengotinib in Advanced Solid Tumors: Results from a Multicenter Phase Ib/II Trial
Source: Clin Cancer Res. 2026 Jul 23;32(21):4874–84. doi: 10.1158/1078-0432.CCR-26-1076 (PMC13725326; doi:10.1158/1078-0432.CCR-26-1076)
Supplement: Supplementary file 10 [file ccr-26-1076_supplementary_table_s2_suppts2.pdf]

Supplementary Table S2. Gene List for the FoundationOne® Liquid CDx Assay

Gene List						
ABL1	*ACVR1B*	***AKT1***	*AKT2*	*AKT3*	***ALK***	*ALOX12B*
AMER1 (*FAM123B*)	***APC***	***AR***	***ARAF***	*ARFRP1*	*ARID1A*	*ASXL1*
ATM	***ATR***	*ATRX*	*AURKA*	*AURKB*	*AXIN1*	*AXL*
BAP1	*BARD1*	*BCL2*	*BCL2L1*	*BCL2L2*	*BCL6*	*BCOR*
BCORL1	*BCR**	***BRAF***	***BRCA1***	***BRCA2***	*BRD4*	*BRIP1*
BTG1	*BTG2*	***BTK***	*C11orf30* (*EMSY*)	*C17orf39* (*GID4*)	*CALR*	*CARD11*
CASP8	*CBFB*	*CBL*	***CCND1***	*CCND2*	*CCND3*	*CCNE1*
CD22	*CD70*	*CD74**	*CD79A*	*CD79B*	***CD274*** **(*PD-L1*)**	*CDC73*
CDH1	***CDK12***	***CDK4***	***CDK6***	*CDK8*	*CDKN1A*	*CDKN1B*
CDKN2A	*CDKN2B*	*CDKN2C*	*CEBPA*	*CHEK1*	***CHEK2***	*CIC*
CREBBP	***CRKL***	*CSF1R*	*CSF3R*	*CTCF*	*CTNNA1*	***CTNNB1***
CUL3	*CUL4A*	*CXCR*	*CYP17A1*	*DAXX*	*DDR1*	***DDR2***
DIS3	*DNMT3A*	*DOT1L*	*EED*	***EGFR***	*EP300*	*EPHA3*
EPHB1	*EPHB4*	***ERBB2***	***ERBB3***	*ERBB4*	*ERCC4*	*ERG*
ERRFI1	***ESR1***	*ETV4**	*ETV5**	***ETV6****	*EWSR1**	***EZH2***
*EZR**	*FAM46C*	*FANCA*	*FANCC*	*FANCG*	*FANCL*	*FAS*
FBXW7	*FGF10*	*FGF12*	*FGF14*	*FGF19*	*FGF23*	*FGF3*
FGF4	*FGF6*	***FGFR1***	***FGFR2***	***FGFR3***	*FGFR4*	*FH*
FLCN	*FLT1*	***FLT3***	***FOXL2***	*FUBP1*	*GABRA6*	*GATA3*
GATA4	*GATA6*	***GNA11***	*GNA13*	***GNAQ***	***GNAS***	*GRM3*
GSK3B	*H3F3A*	*HDAC1*	*HGF*	*HNF1A*	***HRAS***	*HSD3B1*
ID3	***IDH1***	***IDH2***	*IGF1R*	*IKBKE*	*IKZF1*	*INPP4B*
IRF2	*IRF4*	*IRS2*	*JAK1*	***JAK2***	***JAK3***	*JUN*
KDM5A	*KDM5C*	*KDM6A*	*KDR*	*KEAP1*	*KEL*	***KIT***
KLHL6	*KMT2A* (*MLL*)	*KMT2D* (*MLL2*)	***KRAS***	*LTK*	*LYN*	*MAF*
MAP2K1 **(*MEK1*)**	***MAP2K2*** **(*MEK2*)**	*MAP2K4*	*MAP3K1*	*MAP3K13*	*MAPK1*	*MLC1*
MDM2	*MDM4*	*MED12*	*MEF2B*	*MEN1*	*MERTK*	***MET***
MITF	*MKNK1*	*MLH1*	***MPL***	*MRE11A*	*MSH2*	*MSH3*
MSH6	*MST1R*	*MTAP*	***MTOR***	*MUTYHMYB**	***MYC***	*MYCL* (*MYCL1*)
MYCN	***MYD88***	*NBN*	*NF1*	*NF2*	*NFE2L2*	*NFKBIA*
NKX2-1	*NOTCH1*	*NOTCH2*	*NOTCH3*	***NPM1***	***NRAS***	*NSD3* (*WHSC1L1*)
NT5C2	***NTRK1***	*NTRK2*	***NTRK3***	*NUTM1**	*P2RYB*	***PALB2***
PARK2	*PARP1*	*PARP2*	*PARP3*	*PAX5*	*PBRM1*	
PDCD1 (*PD-1*)	***PDCD1LG2*** **(*PD-L2*)**	***PDGFRA***	***PDGFRB***	*PDK1*	*PIK3C2B*	*PIK3C2G*

Gene List						
PIK3CA	*PIK3CB*	*PIK3R1*	*PIM1*	*PMS2*	*POLD1*	*POLE*
PPARG	*PPP2R1A*	*PPP2R2A*	*PRDM1*	*PRKAR1A*	*PRKCI*	*PTCH1*
PTEN	***PTPN11***	*PTPRO*	*QKI*	*RAC1*	*RAD21*	*RAD51*
RAD51B	*RAD51C*	*RAD51D*	*RAD52*	*RAD54L*	***RAF1***	*RARA*
RB1	*RBM10*	*REL*	*RET*	*RICTOR*	*RNF43*	***ROS1***
RPTOR	*RSPO2**	*SDC4**	*SDHA*	*SDHB*	*SDHC*	*SDHD*
SETD2	*SF3B1*	*SGK1*	*SLC34A2**	*SMAD2*	*SMAD4*	*SMARCA4*
SMARCB1	***SMO***	*SNCAIP*	*SOCS1*	*SOX2*	*SOX9*	*SPEN*
SPOP	*SRC*	*STAG2*	*STAT3*	***STK11***	*SUFU*	*SYK*
TBX3	*TEK*	*TERC** noncoding RNA	***TERT**** **promoter**	*TET2*	*TGFBR2*	*TIPARP*
*TMPRSS2**	*TNFAIP3*	*TNFRSF14*	***TP53***	*TSC1*	*TSC2*	*TYRO3*
U2AF1	***VEGFA***	*VHL*	*WHSC1*	*WT1*	*XPO1*	*XRCC2*
ZNF217	*ZNF703*					

Note: This assay was used to detect genomic alterations and to identify patients who may benefit from treatment with targeted therapies. The assessment interrogates 324 genes, including 309 genes with complete exonic (coding) coverage and 15 genes with only select non-coding coverage (indicated with an *); 75 genes (**indicated in bold**) are captured with increased sensitivity. More details on the test can be found at https://www.foundationmedicine.com/.
