# Supplementary material for: Multikinase Inhibition with Tinengotinib in Advanced Solid Tumors: Results from a Multicenter Phase Ib/II Trial
Source: Clin Cancer Res. 2026 Jul 23;32(21):4874–84. doi: 10.1158/1078-0432.CCR-26-1076 (PMC13725326; doi:10.1158/1078-0432.CCR-26-1076)
Supplement: Supplementary file 11 [file ccr-26-1076_supplementary_table_s3_suppts3.pdf]

Table 3. Tumor Response Assessments per Dosage Group (Efficacy Analysis Set)

n (%)	Arm C					Arms A + C	
	**4 mg BID (n=15)**	**6 mg BID (n=18)**	**5 mg QD (n=10)**	**8 mg QD (n=13)**	**10 mg QD (n=19)**	**12 mg QD (n=84)**	**Overall (N=159)**
ORR	0	2 (11.1)	1 (10.0)	0	4 (21.1)	14 (16.7)	21 (13.2)
CR	0	0	0	0	0	1 (1.2)	1 (0.6)
PR	0	2 (11.1)	1 (10.0)	0	4 (21.1)	13 (15.5)	20 (12.6)
SD	7 (46.7)	6 (33.3)	2 (20.0)	6 (46.2)	10 (52.6)	41 (48.8)	72 (45.3)
SD ≥6 months	3 (20.0)	1 (5.6)	0	4 (30.8)	3 (15.8)	11 (13.1)	22 (13.8)
PD	8 (53.3)	9 (50.0)	6 (60.0)	7 (53.8)	4 (21.1)	23 (27.4)	57 (35.8)
Not evaluable	0	1 (5.6)	1 (10.0)	0	1 (5.3)	6 (7.1)	9 (5.7)
DCR[a] [95% CI]	7 (46.7) [21.3–73.4]	8 (44.4) [21.5–69.2]	3 (30.0) [6.7–65.2]	6 (46.2) [19.2–74.9]	14 (73.7) [48.8–90.9]	55 (65.5) [54.3–75.5]	93 (58.5) [50.4–66.2]
DOR, events[b]	0	1	1	0	3	11	16
DOR estimate,[c] months, median [95% CI]	NA [NA–NA]	8.3 [NA–NA]	9.5 [NA–NA]	NA [NA–NA]	2.5 [0.1–NA]	4.2 [1.7–7.7]	4.2 [1.7–8.3]

Note: Data are shown as n (%) unless otherwise noted.

[a]DCR = CR + PR + SD.

[b]Events included progression or death.

[c]Calculated using the Kaplan-Meier estimator. 95% CIs were calculated using log-log transformation.

Abbreviations: BID, twice daily; CI, confidence interval; CR, complete response; DCR, disease control rate; DOR, duration of response; NA, not available; ORR, objective response rate; PD, progressive disease; PR, partial response; QD, once daily; SD, stable disease.
