# Supplementary material for: Multikinase Inhibition with Tinengotinib in Advanced Solid Tumors: Results from a Multicenter Phase Ib/II Trial
Source: Clin Cancer Res. 2026 Jul 23;32(21):4874–84. doi: 10.1158/1078-0432.CCR-26-1076 (PMC13725326; doi:10.1158/1078-0432.CCR-26-1076)
Supplement: Supplementary file 12 [file ccr-26-1076_supplementary_table_s4_suppts4.pdf]

Supplementary Table S4. Prior Lines of Therapies for Cholangiocarcinoma, Breast Cancer, and Prostate Cancer Populations as well as Total Population (A) and Baseline FGFR Status (B).

A

**Baseline characteristics**	**CCA (n=27)**	**Breast cancer (n=21)**	**Prostate cancer (n=17)**	**Total population (N=197)[a]**
Prior line of therapies				
1	4 (14.8)	1 (4.8)	2 (11.8)	15 (7.6)
2	7 (25.9)	2 (9.5)	1 (5.9)	34 (17.3)
≥3	16 (59.3)	18 (85.7)	14 (82.4)	145 (73.6)
FGFR inhibitor type				
Pemigatinib	3 (11.1)	0	0	5 (2.5)*
Lirafugratinib	2 (7.4)	0	0	2 (1.0)

B

	**Arm A**	**Arm C**						**Arms A + C**	
**Characteristic**	**12 mg QD (n=86)**	**4 mg BID (n=18)**	**6 mg BID (n=18)**	**5 mg QD (n=14)**	**8 mg QD (n=18)**	**10 mg QD (n=21)**	**12 mg QD (n=22)**	**12 mg QD (n=108)**	**Overall (N=197)**
FGFR status									
FGFR2 fusion	4 (4.7)	0	1 (5.6)	0	0	0	2 (9.1)	6 (5.6)	7 (3.6)
FGFR2 fusion, FGFR2 rearrangement	1 (1.2)	0	0	0	0	0	0	1 (0.9)	1 (0.5)
FGFR2 mutation	1 (1.2)	2 (11.1)	1 (5.6)	3 (21.4)	1 (5.6)	0	0	1 (0.9)	8 (4.1)
FGFR2 rearrangement	2 (2.3)	0	0	0	0	0	1 (4.5)	3 (2.8)	3 (1.5)
No documented FGFR alternation	65 (75.6)	14 (77.8)	15 (83.3)	10 (71.4)	17 (94.4)	21 (100)	18 (81.8)	83 (76.9)	160 (81.2)
Other	13 (15.1)	2 (11.1)	1 (5.6)	1 (7.1)	0	0	1 (4.5)	14 (13.0)	18 (9.1)

[a]Prior lines of therapies were missing for three patients.
*CCA, n=3; sarcoma, n=1; and other indication, n=1.
CCA, cholangiocarcinoma; FGFR, fibroblast growth factor receptors.
