# Supplementary material for: Multikinase Inhibition with Tinengotinib in Advanced Solid Tumors: Results from a Multicenter Phase Ib/II Trial
Source: Clin Cancer Res. 2026 Jul 23;32(21):4874–84. doi: 10.1158/1078-0432.CCR-26-1076 (PMC13725326; doi:10.1158/1078-0432.CCR-26-1076)
Supplement: Supplementary file 13 [file ccr-26-1076_supplementary_table_s5_suppts5.pdf]

Supplementary Table S5. Representativeness of Study Participants

Cancer type	Advanced solid tumors (including but not limited to cholangiocarcinoma, breast cancer, prostate cancer, and other refractory solid tumors).
Considerations related to:	
Sex	According to the 2024 Annual Report to the Nation on the Status of Cancer, the reported rate of new cancer cases is higher among men versus women in the United States.
Age	In the United States, the median age at diagnosis for many solid tumors is approximately 60–70 years, consistent with the population enrolled in this study.
Race/ethnicity	Based on analyses of US clinical trials, patients are typically predominantly White (>50%), with lower representation of minority populations.
Geography	According to U.S. Cancer Statistics, solid tumor incidence is reported for every state, and the burden differs across the country. Some states (Midwest and South) tend to have higher incidence of certain solid tumors (especially lung and colorectal cancers). Western states generally have lower rates of several solid tumors (breast, lung), partly due to population risk factor patterns.
Other considerations	Advanced solid tumors (including but not limited to cholangiocarcinoma, breast cancer, prostate cancer, and other refractory solid tumors) are common in the United States.
Overall representativeness of this study	The study population is representative of patients with advanced, treatment-refractory solid tumors treated in United States clinical settings. Overall, 43.7% of patients were male and 56.3% were female. The higher proportion of female patients likely reflects the inclusion of tumor types such as breast and gynecologic cancers. The median age of patients in this study was 61 years and the majority of patients were White (71.1%) followed by Black/African American (12.2%), Asian (7.6%), and other racial/ethnic groups.<br>The inclusion of multiple tumor types and prior lines of therapy reflects real-world oncology practice; however, geographic restriction to the United States may limit generalizability to other populations.
