# Supplementary material for: Multikinase Inhibition with Tinengotinib in Advanced Solid Tumors: Results from a Multicenter Phase Ib/II Trial
Source: Clin Cancer Res. 2026 Jul 23;32(21):4874–84. doi: 10.1158/1078-0432.CCR-26-1076 (PMC13725326; doi:10.1158/1078-0432.CCR-26-1076)
Supplement: Supplementary file 14 [file ccr-26-1076_supplementary_table_s6_suppts6.pdf]

Supplementary Table S6. Summary of TEAEs by Dosage Group (Safety Analysis Set)

	Arm A	Arm C						Arms A + C	
**n (%)**	**12 mg QD (n=86)**	**4 mg BID (n=18)**	**6 mg BID (n=18)**	**5 mg QD (n=14)**	**8 mg QD (n=18)**	**10 mg QD (n=21)**	**12 mg QD (n=22)**	**12 mg QD (N=108)**	**Overall (N=197)**
TEAEs	83 (96.5)	17 (94.4)	18 (100)	12 (85.7)	18 (100)	21 (100)	22 (100)	105 (97.2)	191 (97.0)
Treatment-related TEAEs	71 (82.6)	14 (77.8)	16 (88.9)	7 (50.0)	16 (88.9)	18 (85.7)	18 (81.8)	89 (82.4)	160 (81.2)
Serious TEAEs	45 (52.3)	6 (33.3)	12 (66.7)	7 (50.0)	9 (50.0)	14 (66.7)	11 (50.0)	56 (51.9)	104 (52.8)
Treatment-related serious TEAEs	7 (8.1)	2 (11.1)	5 (27.8)	0	2 (11.1)	5 (23.8)	4 (18.2)	11 (10.2)	25 (12.7)
TEAEs by maximum severity									
Grade 1	3 (3.5)	5 (27.8)	2 (11.1)	0	3 (16.7)	1 (4.8)	0	3 (2.8)	14 (7.1)
Grade 2	13 (15.1)	4 (22.2)	1 (5.6)	2 (14.3)	4 (22.2)	5 (23.8)	4 (18.2)	17 (15.7)	33 (16.8)
Grade 3	61 (70.9)	8 (44.4)	13 (72.2)	7 (50.0)	9 (50.0)	12 (57.1)	16 (72.7)	77 (71.3)	126 (64.0)
Grade 4	2 (2.3)	0	2 (11.1)	3 (21.4)	1 (5.6)	2 (9.5)	0	2 (1.9)	10 (5.1)
Grade 5	4 (4.7)	0	0	0	1 (5.6)	1 (4.8)	2 (9.1)	6 (5.6)	8 (4.1)
Treatment-related TEAE by maximum severity									
Grade 1	7 (8.1)	6 (33.3)	3 (16.7)	1 (7.1)	4 (22.2)	3 (14.3)	1 (4.5)	8 (7.4)	25 (12.7)
Grade 2	21 (24.4)	5 (27.8)	3 (16.7)	0	5 (27.8)	4 (19.0)	7 (31.8)	28 (25.9)	45 (22.8)
Grade 3	43 (50.0)	3 (16.7)	9 (50.0)	6 (42.9)	7 (38.9)	9 (42.9)	10 (45.5)	53 (49.1)	87 (44.2)
Grade 4	0	0	1 (5.6)	0	0	2 (9.5)	0	0	3 (1.5)
TEAEs leading to study drug discontinuation	23 (26.7)	2 (11.1)	7 (38.9)	3 (21.4)	2 (11.1)	5 (23.8)	6 (27.3)	29 (26.9)	48 (24.4)

	Arm A	Arm C					Arms A + C	
n (%)	12 mg QD (n=86)	4 mg BID (n=18)	6 mg BID (n=18)	5 mg QD (n=14)	8 mg QD (n=18)	10 mg QD (n=21)	12 mg QD (n=22)	12 mg QD (N=108)	Overall (N=197)
Treatment-related TEAEs leading to study drug discontinuation	9 (10.5)	0	2 (11.1)	0	1 (5.6)	3 (14.3)	2 (9.1)	11 (10.2)	17 (8.6)
TEAEs leading to death	4 (4.7)	0	0	0	1 (5.6)	1 (4.8)	2 (9.1)	6 (5.6)	8 (4.1)
Treatment-related TEAEs leading to death	0	0	0	0	0	0	0	0	0
Most common TEAEs									
Hypertension	50 (58.1)	5 (27.8)	8 (44.4)	3 (21.4)	9 (50.0)	11 (52.4)	13 (59.1)	63 (58.3)	99 (50.3)
Grade ≥3	16 (18.6)	1 (5.6)	4 (22.2)	3 (21.4)	3 (16.7)	5 (23.8)	3 (13.6)	19 (17.6)	35 (17.8)
Diarrhea	28 (32.6)	7 (38.9)	9 (50.0)	3 (21.4)	9 (50.0)	7 (33.3)	12 (54.5)	40 (37.0)	75 (38.1)
Grade ≥3	3 (3.5)	2 (11.1)	2 (11.1)	1 (7.1)	1 (5.6)	1 (4.8)	2 (9.1)	5 (4.6)	12 (6.1)
Stomatitis	32 (37.2)	3 (16.7)	8 (44.4)	2 (14.3)	5 (27.8)	8 (38.1)	10 (45.5)	42 (38.9)	68 (34.5)
Grade ≥3	10 (11.6)	0	3 (16.7)	0	0	1 (4.8)	3 (13.6)	13 (12.0)	17 (8.6)
Nausea	22 (25.6)	6 (33.3)	8 (44.4)	5 (35.7)	10 (55.6)	7 (33.3)	9 (40.9)	31 (28.7)	67 (34.0)
Grade ≥3	2 (2.3)	0	2 (11.1)	0	2 (11.1)	1 (4.8)	0	2 (1.9)	7 (3.6)
Vomiting	17 (19.8)	8 (44.4)	8 (44.4)	5 (35.7)	8 (44.4)	8 (38.1)	7 (31.8)	24 (22.2)	61 (31.0)
Grade ≥3	2 (2.3)	0	1 (5.6)	1 (7.1)	2 (11.1)	1 (4.8)	0	2 (1.9)	7 (3.6)

	Arm A	Arm C						Arms A + C	
	**12 mg QD**	**4 mg BID**	**6 mg BID**	**5 mg QD**	**8 mg QD**	**10 mg QD**	**12 mg QD**	**12 mg QD**	**Overall**
**n (%)**	**(n=86)**	**(n=18)**	**(n=18)**	**(n=14)**	**(n=18)**	**(n=21)**	**(n=22)**	**(N=108)**	**(N=197)**
Fatigue	25 (29.1)	5 (27.8)	7 (38.9)	3 (21.4)	7 (38.9)	6 (28.6)	3 (13.6)	28 (25.9)	56 (28.4)
Grade ≥3	4 (4.7)	0	1 (5.6)	0	0	2 (9.5)	1 (4.5)	5 (4.6)	8 (4.1)
Decreased appetite	18 (20.9)	4 (22.2)	5 (27.8)	3 (21.4)	4 (22.2)	10 (47.6)	6 (27.3)	24 (22.2)	50 (25.4)
Grade ≥3	1 (1.2)	0	0	0	1 (5.6)	1 (4.8)	0	1 (0.9)	3 (1.5)
Constipation	24 (27.9)	3 (16.7)	4 (22.2)	5 (35.7)	3 (16.7)	2 (9.5)	5 (22.7)	29 (26.9)	46 (23.4)
Grade ≥3	2 (2.3)	1 (5.6)	0	0	0	0	0	2 (1.9)	3 (1.5)
Abdominal pain	17 (19.8)	2 (11.1)	3 (16.7)	5 (35.7)	5 (27.8)	6 (28.6)	6 (27.3)	23 (21.3)	44 (22.3)
Grade ≥3	8 (9.3)	1 (5.6)	2 (11.1)	1 (7.1)	3 (16.7)	2 (9.5)	3 (13.6)	11 (10.2)	20 (10.2)
PPE syndrome	20 (23.3)	3 (16.7)	3 (16.7)	2 (14.3)	3 (16.7)	7 (33.3)	6 (27.3)	26 (24.1)	44 (22.3)
Grade ≥3	8 (9.3)	1 (5.6)	1 (5.6)	1 (7.1)	0	0	1 (4.5)	9 (8.3)	12 (6.1)

Abbreviations: BID, twice daily; PPE, palmar-plantar erythrodysesthesia; QD, once daily; TEAE, treatment-emergent adverse event.
