# Supplementary material for: Multikinase Inhibition with Tinengotinib in Advanced Solid Tumors: Results from a Multicenter Phase Ib/II Trial
Source: Clin Cancer Res. 2026 Jul 23;32(21):4874–84. doi: 10.1158/1078-0432.CCR-26-1076 (PMC13725326; doi:10.1158/1078-0432.CCR-26-1076)
Supplement: Supplementary file 15 [file ccr-26-1076_supplementary_table_s7_suppts7.pdf]

Supplementary Table S7. Most Common TEAEs and Severity by Dosage Group (Safety Analysis Set)

	Arm A	Arm C						Arms A + C	
**Most Common TEAEs, n (%)**	**12 mg QD (n=86)**	**4 mg BID (n=18)**	**6 mg BID (n=18)**	**5 mg QD (n=14)**	**8 mg QD (n=18)**	**10 mg QD (n=21)**	**12 mg QD (n=22)**	**12 mg QD (n=108)**	**Overall (N=197)**
Hypertension	50 (58.1)	5 (27.8)	8 (44.4)	3 (21.4)	9 (50.0)	11 (52.4)	13 (59.1)	63 (58.3)	99 (50.3)
Grades 1–2	34 (39.5)	4 (22.2)	4 (22.2)	0	6 (33.3)	6 (28.6)	10 (45.5)	44 (40.7)	64 (32.5)
Grade 3	16 (18.6)	1 (5.6)	4 (22.2)	3 (21.4)	3 (16.7)	4 (19.0)	3 (13.6)	19 (17.6)	34 (17.3)
Grade 4	0	0	0	0	0	1 (4.8)	0	0	1 (0.5)
Diarrhea	28 (32.6)	7 (38.9)	9 (50.0)	3 (21.4)	9 (50.0)	7 (33.3)	12 (54.5)	40 (37.0)	75 (38.1)
Grade 1–2	25 (29.1)	5 (27.8)	7 (38.9)	2 (14.3)	8 (44.4)	6 (28.6)	10 (45.5)	35 (32.4)	63 (32.0)
Grade 3	3 (3.5)	2 (11.1)	2 (11.1)	1 (7.1)	1 (5.6)	1 (4.8)	2 (9.1)	5 (4.6)	12 (6.1)
Grade 4	0	0	0	0	0	0	0	0	0
Stomatitis	32 (37.2)	3 (16.7)	8 (44.4)	2 (14.3)	5 (27.8)	8 (38.1)	10 (45.5)	42 (38.9)	68 (34.5)
Grades 1–2	22 (25.6)	3 (16.7)	5 (27.8)	2 (14.3)	5 (27.8)	7 (33.3)	7 (31.8)	29 (26.9)	51 (25.9)
Grade 3	10 (11.6)	0	3 (16.7)	0	0	1 (4.8)	3 (13.6)	13 (12.0)	17 (8.6)
Grade 4	0	0	0	0	0	0	0	0	0
Nausea	22 (25.6)	6 (33.3)	8 (44.4)	5 (35.7)	10 (55.6)	7 (33.3)	9 (40.9)	31 (28.7)	67 (34.0)
Grades 1–2	20 (23.3)	6 (33.3)	6 (33.3)	5 (35.7)	8 (44.4)	6 (28.6)	9 (40.9)	29 (26.9)	60 (30.5)
Grade 3	2 (2.3)	0	2 (11.1)	0	2 (11.1)	1 (4.8)	0	2 (1.9)	7 (3.6)
Grade 4	0	0	0	0	0	0	0	0	0
Vomiting	17 (19.8)	8 (44.4)	8 (44.4)	5 (35.7)	8 (44.4)	8 (38.1)	7 (31.8)	24 (22.2)	61 (31.0)
Grades 1–2	15 (17.4)	8 (44.4)	7 (38.9)	4 (28.6)	6 (33.3)	7 (33.3)	7 (31.8)	22 (20.4)	54 (27.4)
Grade 3	2 (2.3)	0	1 (5.6)	1 (7.1)	2 (11.1)	1 (4.8)	0	2 (1.9)	7 (3.6)
Grade 4	0	0	0	0	0	0	0	0	0
Fatigue	25 (29.1)	5 (27.8)	7 (38.9)	3 (21.4)	7 (38.9)	6 (28.6)	3 (13.6)	28 (25.9)	56 (28.4)
Grades 1–2	21 (24.4)	5 (27.8)	6 (33.3)	3 (21.4)	7 (38.9)	4 (19.0)	2 (9.1)	23 (21.3)	48 (24.4)
Grade 3	4 (4.7)	0	1 (5.6)	0	0	2 (9.5)	1 (4.5)	5 (4.6)	8 (4.1)
Grade 4	0	0	0	0	0	0	0	0	0
Decreased appetite	18 (20.9)	4 (22.2)	5 (27.8)	3 (21.4)	4 (22.2)	10 (47.6)	6 (27.3)	24 (22.2)	50 (25.4)
Grades 1–2	17 (19.8)	4 (22.2)	5 (27.8)	3 (21.4)	3 (16.7)	9 (42.9)	6 (27.3)	23 (21.3)	47 (23.9)
Grade 3	1 (1.2)	0	0	0	1 (5.6)	1 (4.8)	0	1 (0.9)	3 (1.5)
Grade 4	0	0	0	0	0	0	0	0	0
Constipation	24 (27.9)	3 (16.7)	4 (22.2)	5 (35.7)	3 (16.7)	2 (9.5)	5 (22.7)	29 (26.9)	46 (23.4)
Grades 1–2	22 (25.6)	2 (11.1)	4 (22.2)	5 (35.7)	3 (16.7)	2 (9.5)	5 (22.7)	27 (25.0)	43 (21.8)
Grade 3	2 (2.3)	1 (5.6)	0	0	0	0	0	2 (1.9)	3 (1.5)
Grade 4	0	0	0	0	0	0	0	0	0
Abdominal pain	17 (19.8)	2 (11.1)	3 (16.7)	5 (35.7)	5 (27.8)	6 (28.6)	6 (27.3)	23 (21.3)	44 (22.3)
Grades 1–2	9 (10.5)	1 (5.6)	1 (5.6)	4 (28.6)	2 (11.1)	4 (19.0)	3 (13.6)	12 (11.1)	24 (12.2)
Grade 3	8 (9.3)	1 (5.6)	2 (11.1)	1 (7.1)	3 (16.7)	2 (9.5)	3 (13.6)	11 (10.2)	20 (10.2)

Most Common TEAEs, n (%)	Arm A 12 mg QD (n=86)	Arm C 4 mg BID (n=18)	6 mg BID (n=18)	5 mg QD (n=14)	8 mg QD (n=18)	10 mg QD (n=21)	12 mg QD (n=22)	Arms A + C 12 mg QD (n=108)	Overall (N=197)
Grade 4	0	0	0	0	0	0	0	0	0
PPE syndrome	20 (23.3)	3 (16.7)	3 (16.7)	2 (14.3)	3 (16.7)	7 (33.3)	6 (27.3)	26 (24.1)	44 (22.3)
Grades 1–2	12 (14.0)	2 (11.1)	2 (11.1)	1 (7.1)	3 (16.7)	7 (33.3)	5 (22.7)	17 (15.7)	32 (16.2)
Grade 3	8 (9.3)	1 (5.6)	1 (5.6)	1 (7.1)	0	0	1 (4.5)	9 (8.3)	12 (6.1)
Grade 4	0	0	0	0	0	0	0	0	0

Note: Adverse events were assigned a severity grade using the Common Terminology Criteria for Adverse Events version 5.0.
Abbreviations: BID, twice daily; PPE, palmar-plantar erythrodysesthesia; QD, once daily; TEAE, treatment-emergent adverse event.
