# Supplementary material for: Multikinase Inhibition with Tinengotinib in Advanced Solid Tumors: Results from a Multicenter Phase Ib/II Trial
Source: Clin Cancer Res. 2026 Jul 23;32(21):4874–84. doi: 10.1158/1078-0432.CCR-26-1076 (PMC13725326; doi:10.1158/1078-0432.CCR-26-1076)
Supplement: Supplementary file 16 [file ccr-26-1076_supplementary_table_s8_suppts8.pdf]

Supplementary Table S8. Tumor Response Assessments per Dosage Group by Tumor Type

Response Assessment by Tumor Type	4 mg BID	6 mg BID	8 mg QD	10 mg QD	12 mg QD	Total
**Patients with CCA**	**n=4**	**n=4**	**n=0**	**n=2**	**n=13**	**N=23**
ORR	0	1 (25.0)	0	0	2 (15.4)	3 (13.0)
PR	0	1 (25.0)	0	0	2 (15.4)	3 (13.0)
SD	2 (50.0)	0	0	0	6 (46.2)	8 (34.8)
SD ⩾6 months	1 (25.0)	0	0	0	1 (7.7)	2 (8.7)
PD	2 (50.0)	3 (75.0)	0	1 (50.0)	4 (30.8)	10 (43.5)
NE	0	0	0	1 (50.0)	1 (7.7)	2 (8.7)
DCR[a] [95% CI]	2 (50.0) [6.8–93.2]	1 (25.0) [0.6–80.6]	0	0	8 (61.5) [31.6–86.1]	11 (47.8) [26.8–69.4]
**Patients with PC**	**n=0**	**n=0**	**n=1**	**n=1**	**n=13**	**N=15**
ORR	—	0	0	1 (100)	4 (30.8)	5 (33.3)
PR	—	0	0	1 (100)	4 (30.8)	5 (33.3)
SD	—	0	0	0	4 (30.8)	4 (26.7)
SD ≥6 months	—	0	0	0	1 (7.7)	1 (6.7)
PD	—	0	1 (100)	0	4 (30.8)	5 (33.3)
NE	—	0	0	0	1 (7.7)	1 (6.7)
DCR[a] [95% CI]	—	0	0	1 (100) [2.5–100]	8 (61.5) [31.6–86.1]	9 (60.0) [32.3–83.7]
**Patients with BC**	**n=0**	**n=2**	**n=0**	**n=3**	**n=15**	**N=20**
ORR	—	0	—	1 (33.3)	5 (33.3)	6 (30.0)
CR	—	0	—	0	1 (6.7)	1 (5.0)
PR	—	0	—	1 (33.3)	4 (26.7)	5 (25.0)
SD	—	2 (100)	—	1 (33.3)	4 (26.7)	7 (35.0)
SD ≥6 months	—	0	—	0	0	0
PD	—	0	—	1 (33.3)	4 (26.7)	5 (25.0)
NE	—	0	—	0	2 (13.3)	2 (10.0)
DCR[a] [95% CI]	—	2 (100) [15.8–100]	—	2 (66.7) [9.4–99.2]	9 (60.0) [32.3–83.7]	13 (65.0) [40.8–84.6]

Note: Data are shown as n (%) unless otherwise noted.

[a]DCR = CR + PR + SD.

Abbreviations: BC, breast cancer; BID, twice daily; CCA, cholangiocarcinoma; CI, confidence interval; CR, complete response; DCR, disease control rate; NE, not evaluable; ORR, objective response rate; PC, prostate cancer; PD, progressive disease; PR, partial response; QD, once daily; SD, stable disease.
