# Supplementary material for: Multikinase Inhibition with Tinengotinib in Advanced Solid Tumors: Results from a Multicenter Phase Ib/II Trial
Source: Clin Cancer Res. 2026 Jul 23;32(21):4874–84. doi: 10.1158/1078-0432.CCR-26-1076 (PMC13725326; doi:10.1158/1078-0432.CCR-26-1076)
Supplement: Supplementary file 17 [file ccr-26-1076_supplementary_table_s9_suppts9.pdf]

Supplementary Table S9. ORR and DCR among Breast Cancer Population

Parameter, n (%) [95%CI]	HR+HER2 negative (n=8)	TNBC (n=7)	aTNBC (n=5)	Total (n=20)
ORR	3 (37.5) [8.5, 75.5]	0	3 (60.0) [14.7, 94.7]	6 (30.0) [11.9, 54.3]
CR	1 (12.5)	0	0	1 (5.0)
PR	2 (25.0)	0	3 (60.0)	5 (25.0)
SD	3 (37.5)	2 (28.6)	2 (40.0)	7 (35.0)
SD >6 months	0	0	0	0
PD	2 (25.0)	3 (42.9)	0	5 (25.0)
NE	0	2 (28.6)	0	2 (10.0)
DCR	6 (75.0) [34.9, 96.8]	2 (28.6) [3.7, 71.0]	5 (100) [47.8, 100]	13 (65.0) [40.8, 84.6]
Clinical benefit rate (CR + PR + SD ≥ 6 months)	3 (37.5) [8.5, 75.5]	0	3 (60.0) [14.7, 94.7]	6 (30.0) [11.9, 54.3]
Duration of response, median [95% CI], months	4.17 [3.48, NA]	0	3.89 [2.92, NA]	4.17 [2.92, NA]

Abbreviations: aTNBC, acquired TNBC; CR, complete response; DCR, disease control rate; NE, not evaluable; ORR, objective response rate; PD, progressive disease; PR, partial response; SD, stable disease; TNBC, triple-negative breast cancer.
